# Pannexin 1 activity in astroglia sets hippocampal neuronal network patterns

**DOI:** 10.1371/journal.pbio.3001891

**Published:** 2022-12-07

**Authors:** Flora Vasile, Elena Dossi, Julien Moulard, Pascal Ezan, Laure Lecoin, Martine Cohen-Salmon, Philippe Mailly, Marc Le Bert, Isabelle Couillin, Alexis Bemelmans, Nathalie Rouach

**Affiliations:** 1 Center for Interdisciplinary Research in Biology, Collège de France, CNRS, INSERM, Labex Memolife, PSL Research University, Paris, France; 2 CNRS UMR7355, Experimental and Molecular Immunology and Neurogenetics, Orleans, France; 3 Université Paris-Saclay, Commissariat à l’Energie Atomique et aux Energies Alternatives, CNRS, MIRCen, Laboratoire des Maladies Neurodégénératives, Fontenay-aux-Roses, France; Duke University Medical Center, UNITED STATES

## Abstract

Astroglial release of molecules is thought to actively modulate neuronal activity, but the nature, release pathway, and cellular targets of these neuroactive molecules are still unclear. Pannexin 1, expressed by neurons and astrocytes, form nonselective large pore channels that mediate extracellular exchange of molecules. The functional relevance of these channels has been mostly studied in brain tissues, without considering their specific role in different cell types, or in neurons. Thus, our knowledge of astroglial pannexin 1 regulation and its control of neuronal activity remains very limited, largely due to the lack of tools targeting these channels in a cell-specific way. We here show that astroglial pannexin 1 expression in mice is developmentally regulated and that its activation is activity-dependent. Using astrocyte-specific molecular tools, we found that astroglial-specific pannexin 1 channel activation, in contrast to pannexin 1 activation in all cell types, selectively and negatively regulates hippocampal networks, with their disruption inducing a drastic switch from bursts to paroxysmal activity. This decrease in neuronal excitability occurs via an unconventional astroglial mechanism whereby pannexin 1 channel activity drives purinergic signaling-mediated regulation of hyperpolarisation-activated cyclic nucleotide (HCN)-gated channels. Our findings suggest that astroglial pannexin 1 channel activation serves as a negative feedback mechanism crucial for the inhibition of hippocampal neuronal networks.

## Abstract 2

Astrocytes have mostly been shown to boost neuronal activity. This study reveals that activity-dependent activation of astroglial pannexin 1 channels inhibits hippocampal neuronal networks by decreasing neuronal excitability via purinergic signaling, uncovering a novel astroglial negative feedback loop mechanism.

## Introduction

Astrocytes regulate neuronal network activity in various contexts [1–4]. They have indeed been reported ex vivo to control synchronisation of hippocampal neuronal activity [2] and cortical UP states [1], and in vivo to regulate cortical slow oscillations [3] and hippocampal gamma oscillations [4]. They also participate to pathological network activities consisting in aberrant bursting or epileptiform activities [2,5]. The underlying mechanisms remain elusive. In particular, which molecules are involved and what are their downstream effects are still open questions.

Pannexin 1 (Px1), first described in the year 2000 and homologous to the invertebrate gap junction protein innexin, belongs to the gap junction protein family. However, Px1 does not form gap junctions, due to cysteine residue glycosylation on their extracellular loop, but forms functional, high-conductance, non-selective transmembrane channels [6]. Px1 is expressed in various brain areas and cell types, including neurons [7], microglia, and astrocytes [8]. Channels formed by Px1 constitute a means of cell-to-cell signalling. Px1-mediated intercellular signalling is achieved via release of molecules in the extracellular space as Px1 channels serve as conduits for ions, neurotransmitters, and metabolites smaller than 1.5 kDa. ATP, D-serine, and glutamate were indeed reported to be released from Px1 channels [8–11]. Px1 channels do not appear to be active at rest, but open in response to positive transmembrane potentials, mechanical stretch, and high extracellular K^+^ and ATP [6].

The functional relevance of Px1 in the central nervous system (CNS) has been mostly investigated regardless of cell type, or in neurons: Px1 was implicated in hippocampal synaptic plasticity such as long-term potentiation [12] and mGluR-mediated burst activity [13], although the Px1 expressing cell type mediating these effects was not identified. Cellular dysregulation caused by injury and pathology such as inflammation, ischemia, and epilepsy was also shown to involve Px1 activation [14–18]. A specific role for neuronal Px1 in neurotransmission was first hinted from the observation that Px1 colocalises with the postsynaptic scaffolding protein PSD-95 [19]. Px1 channels in CA3 hippocampal neurons were then shown to open in conditions of ketogenic metabolism and decrease neuronal excitability [20]. Lastly, it was reported that neuronal Px1 channels are involved in the neuronal activity-dependent motility of resting microglia towards active neurons in the optical tectum of larval zebrafish [21]. However, our knowledge of astroglial Px1 activation and function remains limited, owing to lack of tools for cell-specific dissection of the channel’s functions: to date, astroglial Px1 function in the CNS has been mostly inferred from studies on astroglial cultures and dye uptake assays on brain slices. It was reported that Px1 form channels in astrocytes [22], which open in response to stress [23] and elevated extracellular K^+^ concentrations, and release ATP [9] and D-serine [10]. However, whether and how astroglial Px1 regulates neuronal activity in an integrative, multicellular system still remains unclear. Using an astroglial conditional Px1-deficient transgenic mouse and a model of sustained neuronal population activity, we here show that astroglial Px1 negatively regulates hippocampal neuronal network activity by decreasing neuronal excitability through purinergic signalling-mediated regulation of hyperpolarisation-activated cyclic nucleotide (HCN)-gated channels.

## Results

### Expression and activity-dependent function of Px1 channels in hippocampal astrocytes

**Fig 1:**
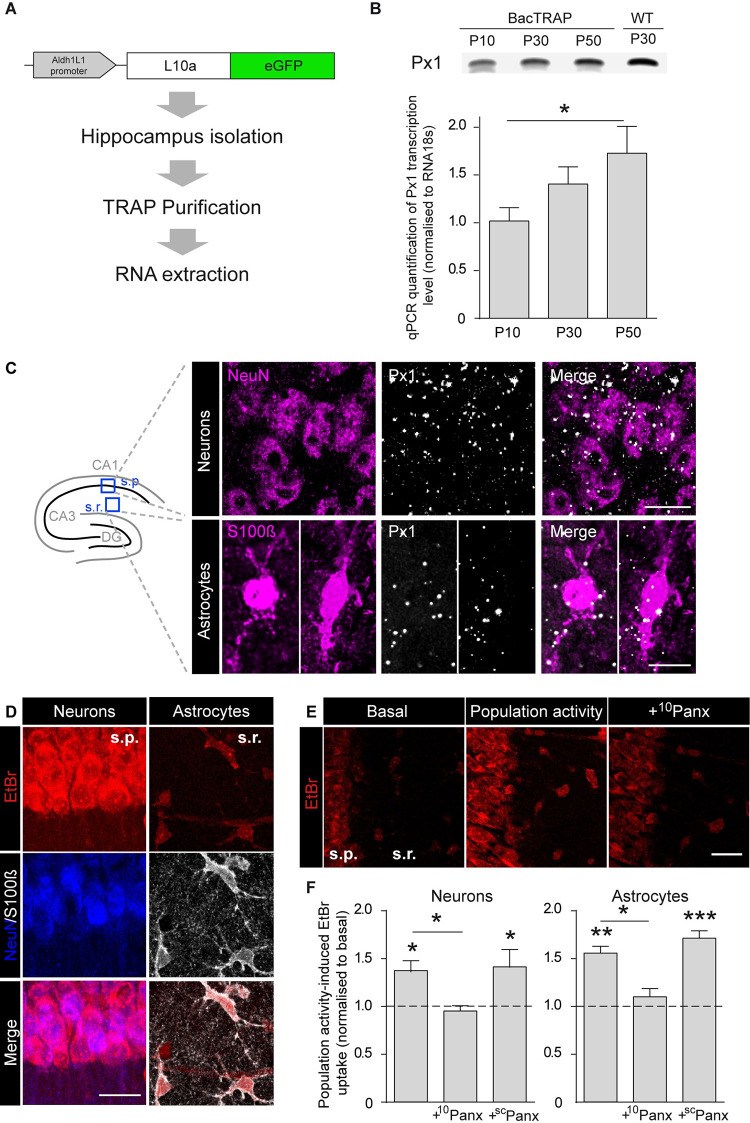
Developmental expression and activity-dependent function of Px1 channels in hippocampal astrocytes. (**A**) Scheme illustrating the protocol used for hippocampal astroglial RNA extraction from Aldh1l1:L10a-eGFP mice using TRAP technique. (**B**) Upper panel: Px1 expression obtained by PCR at different developmental stages (postnatal days 10, 30, and 50) in hippocampal astrocytes (first three lanes) from Aldhl1:L10a-eGFP mice and in total hippocampus (postnatal day 30, last lane). Lower panel: Px1 transcription level normalised to RNA18s quantified by qPCR in P10, P30, and P50 mice in hippocampal astrocytes (*n =* 3 mice; *p* = 0.027; Friedman test followed by Dunn posttest). (**C**) Left: Schematics of the hippocampus showing the CA1 regions of interest from which the representative images are taken. s.p., *stratum pyramidale*; s.r, *stratum radiatum*. Right: Representative confocal images of Px1 mRNA detected in the CA1 region of the hippocampus by FISH by RNAscope on brain sections from P20-P30 C57BL6 mice. Neuron nuclei are immunolabelled with NeuN (top images) and astrocytes with S100β (bottom images). Scale bar, 10 μm. (**D**) Representative EtBr uptake (red) in *stratum pyramidale* (s.p.) neurons immunolabelled with NeuN (blue) and *stratum radiatum* (s.r.) astrocytes immunolabeled with S100β (grey) in hippocampal slices. Scale bar, 20 μm. (**E**) EtBr uptake in basal or population activity conditions without or with ^10^Panx (400 μM) applied 15 min prior and during EtBr uptake assay. Scale bar, 20 μm. (**F**) Quantification of activity-dependent neuronal and astroglial EtBr uptake normalised to control conditions in slices from +/+ mice treated or not with ^10^Panx (neurons, *n =* 9 mice; astrocytes, *n* = 7 mice) and ^sc^Panx (neurons, *n* = 6 mice; astrocytes, *n* = 6 mice; Repeated measures one-way ANOVA). Asterisks indicate statistical significance (*p < 0.05, **p < 0.01). The data underlying this figure can be found in the S1 Metadata A tab.

Consistent with previous reports, we observed Px1 to be strongly expressed in the hippocampus of juvenile mice (P30) (Fig 1A). To investigate expression of Px1 specifically in astrocytes, we first performed PCR on hippocampal astroglial RNA isolated using the TRAP technique on Aldh1l1:L10a-eGFP mice (see Materials and methods; Fig 1B) and found Px1 mRNA expression in astrocytes at various developmental stages, from postnatal days 10 to 50 (P10, P30, P50) (Fig 1A). Next, quantitative PCR (qPCR) revealed that Px1 is developmentally regulated in astrocytes, as its transcription levels were increased at P50 compared to P10 (*n =* 3, *p* = 0.027; Fig 1A). Px1 expression in astrocytes was confirmed by Fluorescent In Situ Hybridisation (FISH), which showed the presence of Px1 mRNAs in both astrocytes (stained with S100β) and neurons (stained with NeuN; *n* = 3 mice; Fig 1C) in wild-type (+/+) mice. We then investigated whether in +/+ mice Px1 channels from hippocampal CA1 astrocytes and pyramidal neurons were functional and their opening activity-dependent. For that purpose, we performed Ethidium Bromide (EtBr) uptake assay in acute hippocampal slices displaying population activity generated spontaneously in a pro-bursting artificial cerebrospinal fluid (ACSF) (Fig 1D and 1E). EtBr being fluorescent and small enough to travel through Px1 channels, the state of opening of these channels can be inferred from fluorescence intensity. In both neurons and astrocytes, population activity significantly increased EtBr uptake compared to basal conditions (neurons, 137 ± 11%, *n =* 9 mice, *p* = 0.0423; astrocytes, 155 ± 7%, *n* = 7 mice, *p* = 0.007; Fig 1E and 1F). This effect was inhibited by blocking selectively Px1 channels with ^10^Panx (400 μM, 15 min) (population activity-induced EtBr uptake normalised to basal ACSF, ^10^Panx: neurons, 95 ± 6%, *n =* 9, *p* = 0.0278; astrocytes, 110 ± 9%, *n* = 7, *p* = 0.0318; Fig 1E and 1F), which resulted in similar EtBr uptake compared to basal condition (*p* = 0.9826 and 0.9103 for neurons and astrocytes, respectively), but not by applying the scramble control peptide ^sc^Panx (400 μM, 15 min) (population activity-induced EtBr uptake normalised to basal ACSF, ^sc^Panx: neurons, 141 ± 19%, *n* = 6, *p* = 0.9933; astrocytes, 171 ± 8%, *n* = 6, *p* = 0.7095; Fig 1F), thus pointing to an activity-dependent uptake through neuronal and astroglial Px1 channels. Consistent with these data, we found using electrophysiology that astroglial Px1 channels are open in population activity regime, since their inhibition by the ^10^Panx peptide in +/+ mice reduced the astrocyte whole-cell conductances recorded at positive potentials, with no change in resting membrane potential or membrane resistance measured at negative membrane potential (S1 Fig; *n =* 4 cells from 3 mice). Altogether, these data indicate an activity-dependent activation of Px1 channels in astrocytes.

### Astroglial Px1 channels limit sustained neuronal population activity

To date, our understanding of Px1 physiological and pathological relevance in the brain mostly relies on genetic and pharmacological disruption of Px1 functions in all brain cells. Here, to investigate the role of astroglial Px1 channels in neuronal network activity, we used molecular approaches, including transgenic mice and viral vectors targeting Px1 in astrocytes. We first engineered a Px1 conditional mutant mouse lacking Px1 expression in astrocytes. To do so, mice with floxed exon 3 (Panx1^tm1c(KOMP)Wtsi^ conditional allele) were crossed with transgenic mice expressing the Cre recombinase under the promoter of the human glial fibrillary acidic protein (hGFAP-cre mice [24]; S2A Fig). To investigate the selectivity and efficiency of Px1 deletion in astrocytes from hGFAP-Cre-Px1^fl/fl^ mice, we first performed FISH using a probe for Px1 exon 3, which is expected to be deleted solely in astrocytes. In hGFAP-Cre-Px1^fl/fl^ mice, while this Px1 probe was detected in the hippocampal pyramidal layer at similar levels compared to +/+ mice (43,969 ± 7,163 versus 55,752 ± 9,104 dots/mm^2^, *p* = 0.3428, *n =* 3 and 3 mice for hGFAP-Cre-Px1^fl/fl^ and +/+ mice; S2B Fig, upper panel), it was significantly reduced in astrocytes (3,520 ± 1,120 versus 22,475 ± 6,698 dots/mm^2^, *p* = 0.0257, *n =* 3 and 3 mice for hGFAP-Cre-Px1^fl/fl^ and +/+ mice; S2B Fig, lower panel), thereby indicating astroglial-specific deletion of Px1.

Consistent with this finding, we found in astroglial Px1-deficient mice a loss of Px1 function selectively in astrocytes, but not in neurons, as assessed by activity-dependent uptake. Indeed, the activity-dependent EtBr uptake induced by sustained network activity was inhibited in astrocytes (population activity-induced EtBr uptake normalised to basal ACSF, 125 ± 12%, *n =* 15 for basal and *n* = 14 for population activity, *p* = 0.2079; S2C and S2D Fig), while it persisted in neurons (139 ± 8%, *n* = 15, *p* = 0.003; S2C and S2D Fig), to a similar level as that found in +/+ mice (*p* = 0.8524; S2D Fig). In addition, inhibiting Px1 channels with the ^10^Panx peptide still significantly decreased EtBr uptake in neurons (106 ± 9%, *p* = 0.0035, *n =* 15; S2D Fig), but not in astrocytes, from hGFAP-Cre-Px1^fl/fl^ mice (117 ± 21%, *p* = 0.3671, *n* = 14; S2D Fig). In contrast, the control ^sc^Panx peptide did not affect activity-dependent EtBr uptake in neurons or astrocytes from hGFAP-Cre-Px1^fl/fl^ mice (neurons, 143 ± 23%, *p* = 0.8450, *n* = 8; astrocytes, 128 ± 23%, *p* = 0.1829, *n* = 8; S2D Fig). Consistent with these data, Px1 disruption in hGFAP-Cre-Px1^fl/fl^ mice decreased astrocyte conductances similarly to Px1 inhibition by ^10^Panx peptide in +/+ mice (S1 Fig; *n =* 5 cells from 3 hGFAP-Cre-Px1^fl/fl^ mice).

Importantly, hGFAP-Cre-Px1^fl/fl^ hippocampi did not present developmental defects. They indeed showed no gross anatomical alterations and presented normal architecture and layered structure, with similar density of CA1 pyramidal cells and astrocytes, as assessed by NeuN and GFAP staining, respectively (S3A and S3B Fig), as well as equivalent *stratum pyramidale* thickness compared to +/+ mice (S3A–S3C Fig; *n =* 9 slices from 3 mice for both +/+ and hGFAP-Cre-Px1^fl/fl^ animals).

**Fig 2:**
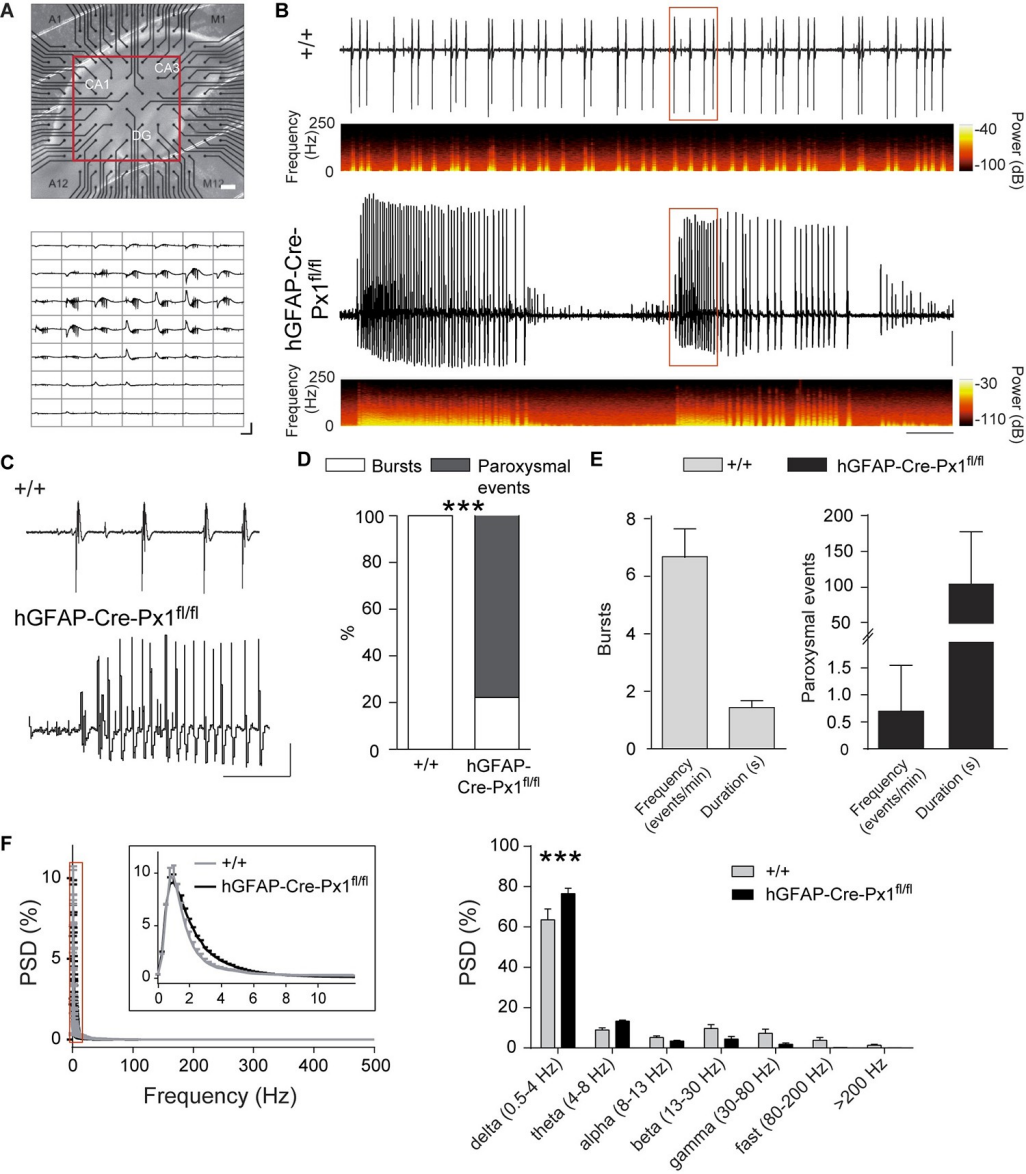
Astroglial Px1 deficiency induces paroxysmal activity. (**A**) Top panel, hippocampal slice on a MEA chamber (DG: dentate gyrus). Bottom panel, illustration of bursting activity recorded using a MEA device (200 μm interelectrode distance) in hippocampal slices. Scale bar: 500 ms, 0.3 mV. (**B**) Representative traces of bursting activity in +/+ mice (upper trace) and paroxysmal activity in hGFAP-Cre-Px1^fl/fl^ mice (lower trace). The corresponding time-frequency plots are shown under the traces. Scale bar: 30 sec, 0.2 mV. (**C**) Magnification of bursting activity in +/+ mice and paroxysmal activity in hGFAP-Cre-Px1^fl/fl^ mice highlighted by the red rectangles in panel B. Scale bar: 10 s, 0.2 mV. (**D**) Proportion of bursts and paroxysmal events recorded in +/+ and hGFAP-Cre-Px1^fl/fl^ mice (+/+, *n =* 13 slices from 6 mice; hGFAP-Cre-Px1^fl/fl^, *n =* 18 slices from 8 mice; Fisher exact test). (**E**) Quantification of bursts and paroxysmal events frequency and duration (+/+, *n* = 13 slices; hGFAP-Cre-Px1^fl/fl^, *n* = 14 slices). (**F**) Left, power spectral density of activity (normalised to the percent of the total PSD) recorded in +/+ (grey) and hGFAP-Cre-Px1^fl/fl^ (black) slices. The area highlighted by the red rectangle is zoomed in the inset. Right, power spectral density of left panel binned according to different brain rhythms: delta (0.5–4 Hz), theta (4–8 Hz), alpha (8–3 Hz), beta (13–30 Hz), gamma (30–80 Hz), fast (80–200 Hz), and >200 Hz oscillations (+/+, *n* = 13 slices; hGFAP-Cre-Px1^fl/fl^, *n* = 12 slices; *p* = 0.002, repeated measures two-way ANOVA). Asterisks indicate statistical significance (*p < 0.05; ***p < 0.0001). The data underlying this figure can be found in the S1 Metadata B tab.

Using this transgenic mouse, we investigated the role of astroglial Px1 channels in neuronal population activity. To do so, we recorded population activity generated spontaneously in a pro-bursting ACSF in hippocampal slices from +/+ and hGFAP-Cre-Px1^fl/fl^ mice using the Multi-Electrode Array (MEA) technique [2] (Fig 2A). Hippocampal slices from +/+ mice exhibited spontaneous bursts with an incidence of 6.69 ± 1.01 bursts/min and a duration of 1.43 ± 0.07 s (*n =* 13 slices from 6 mice; Fig 2B–2D). Strikingly, disrupting astroglial Px1 switched the pattern of spontaneous discharges to paroxysmal events in 77.8% of recorded slices (*p* < 0.001; Fig 2D; paroxysmal events frequency: 0.70 ± 0.22 events/min; duration: 105.22 ± 19.27 s; *n* = 14 out of 18 slices from 8 mice; Fig 2E) and increased delta (0.5 to 4 Hz) activity (*p* < 0.001; Fig 2F). In addition, 41.6% of hGFAP-Cre-Px1^fl/fl^ slices with paroxysmal events displayed occasional interparoxysmal event bursts.

Recombination driven by the hGFAP promoter is not necessarily restricted to astrocytes in hGFAP-Cre-Px1^fl/fl^ mice, due to transient expression of GFAP in other cell types during development. We thus tested whether the switch from bursting to paroxysmal activity observed in hGFAP-Cre-Px1^fl/fl^ animals was also present in conditional and inducible Px1 knockout mice (hGFAP-CreERT2-Px1^fl/fl^), where Px1 deletion is induced postnatally specifically in astrocytes. In these mice, in which tamoxifen (TF)-induced CreERT2 expression leads to recombination in 58.6 ± 2% of GFAP-expressing cells (*n =* 6 slices from 2 mice; S4A Fig), Px1 expression in neurons assessed by FISH is comparable to that observed in +/+ and hGFAP-Cre-Px1^fl/fl^ mice (44,311 ± 9,455 dots/mm^2^; *p* = 0.7192 and *p* > 0.9999 in comparison with +/+ and hGFAP-Cre-Px1^fl/fl^ mice), while it is strongly reduced in astrocytes (6,739 ± 1,308 dots/mm^2^; *p* = 0.0353), to similar levels as in hGFAP-Cre-Px1^fl/fl^ mice (*p* = 0.9372; *n* = 3, 3, and 3 for +/+, hGFAP-Cre-Px1^fl/fl^ and hGFAP-CreERT2-Px1^fl/fl^ mice, respectively; S4B Fig). MEA recordings of slices from hGFAP-CreERT2-Px1^fl/fl^ revealed the same pattern of activity as the one observed in hGFAP-Cre-Px1^fl/fl^ mice (*p* = 0.4569; S4 Fig). Indeed, the majority of slices (62.5%; *n =* 10 out of 16 slices from 4 mice) displayed paroxysmal activity, while bursting activity was observed in only 37.5% of the slices (*n* = 6 out of 16 from 4 mice). Furthermore, paroxysmal activity recorded in slices from hGFAP-CreERT2-Px1^fl/fl^ mice displayed similar frequency and duration compared to hGFAP-Cre-Px1^fl/fl^ slices (frequency: 0.39 ± 0.12 /min, *p* = 0.312; duration: 68.09 ± 12 s, *p* = 0.156; S1 Table). In contrast, control mice (+/+ and hGFAP-CreERT2 treated with TF) mostly displayed bursting activity (86%, *n* = 12 out of 14 slices from 3 mice for +/+ + TF; 77%, *n* = 20 out of 26 slices from 4 mice for hGFAP-CreERT2 + TF; *p* = 0.0106 and 0.027, respectively; S4C–S4G Fig). These results thus indicate that postnatal deletion of Px1 specifically in astrocytes limits neuronal population activity.

Px1 is expressed in both neurons and astrocytes. To evaluate the potential differential role of neuronal versus astroglial Px1, we then compared the effect of global versus astroglial Px1 disruption on neuronal population activity. To assess the effect of global Px1 inhibition, we used either the ^10^Panx peptide in +/+ mice (S5A and S5B Fig) or a constitutive Px1^−/−^ mouse (S5C–S5E Fig), in which Px1 is deleted both in neurons and in astrocytes, as assessed by FISH (neurons: 13,395 ± 4,238 dots/mm^2^; astrocytes: 3,211 ± 1,215 dots/mm^2^; *p* = 0.0027 and 0.0057 for neurons and astrocytes, respectively; *n =* 3 Px1^−/−^ and 3 +/+ mice; S5C Fig). ^10^Panx peptide, as well as Px1 deficiency in constitutive Px1^−/−^ mice, did not induce paroxysmal activity and had no effect on neuronal bursting pattern compared to control condition in +/+ mice (Control (before ^10^Panx): frequency, 7.72 ± 2.45 bursts/min; duration, 1.94 ± 0.25 s; ^10^Panx: frequency, 7.91 ± 2.91 bursts/min, *p* = 0.9193; duration, 2.74 ± 1.01 s, *p* = 0.4770, *n* = 5 slices from 3 mice; S5B Fig; +/+: frequency, 6.68 ± 1.01 bursts/min; duration, 1.43 ± 0.07 s; *n* = 13 slices from 6 mice; Constitutive Px1^−/−^ mice: frequency, 6.90 ± 0.89 bursts/min, *p* = 0.87; duration, 1.25 ± 0.10 s; *n* = 16 slices from 6 mice; *p* = 0.100; S5E Fig). Altogether, these data indicate that ubiquitous Px1 deletion has no effect on activity pattern and suggest that astroglial Px1 differentially regulate network activity compared to neuronal Px1.

**Fig 3:**
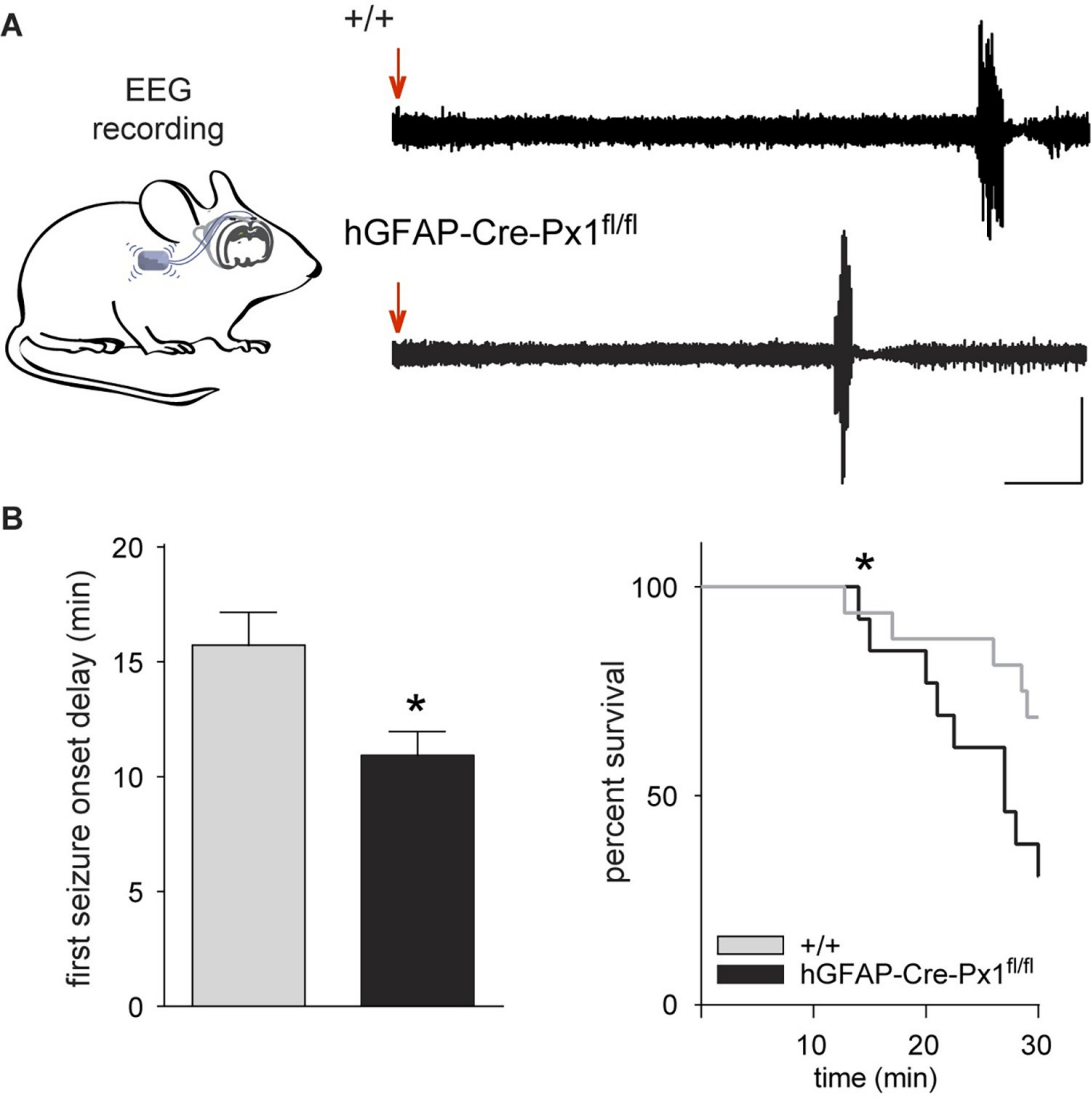
Astroglial Px1-deficient mice are more susceptible to pilocarpine-induced seizures. (**A**) Left, schematic representation of a mouse implanted with wireless ETA-F10 transmitters and EEG electrodes for EEG recordings. Right, representative traces of EEG recordings in +/+ (upper trace) and hGFAP-Cre-Px1^fl/fl^ mice (lower trace). Red arrows indicate pilocarpine injection. Scale bar: 2 min, 500 μV. (**B**) Quantification of first seizure onset delay and percent survival (+/+, *n* = 16 mice; hGFAP-Cre-Px1^fl/fl^, *n* = 13 mice; Student *t* test and probability of survival analysis with log-rank (Mantel–Cox) test). Asterisks indicate statistical significance (*p < 0.05). The data underlying this figure can be found in the S1 Metadata C tab.

Paroxysmal activity recorded in hippocampal slices from hGFAP-Cre-Px1^fl/fl^ mice could translate in vivo into increased susceptibility to seizures. To investigate this, we performed recordings of electroencephalogram (EEG) after intraperitoneal (IP) injection of the proconvulsant agent pilocarpine and found that hGFAP-Cre-Px1^fl/fl^ mice display increased seizure susceptibility in vivo. Indeed, hGFAP-Cre-Px1^fl/fl^ mice had a shorter first seizure onset delay (+/+: 15.73 ± 1.42 min; hGFAP-Cre-Px1^fl/fl^: 10.94 ± 1.04 min; *n =* 16 and 13 mice for +/+ and hGFAP-Cre-Px1^fl/fl^, respectively; *p* = 0.0136; Fig 3A and 3B) and a lower survival rate (+/+: 68.75%; hGFAP-Cre-Px1^fl/fl^ 30.77%; *n =* 16 and 13 mice for +/+ and hGFAP-Cre-Px1^fl/fl^ respectively; *p* = 0.0426; Fig 3B) compared to +/+ mice.

In all, these data show that astroglial Px1 channels inhibit paroxysmal activity.

### Astroglial Px1 channels control excitability of pyramidal cells

**Fig 4:**
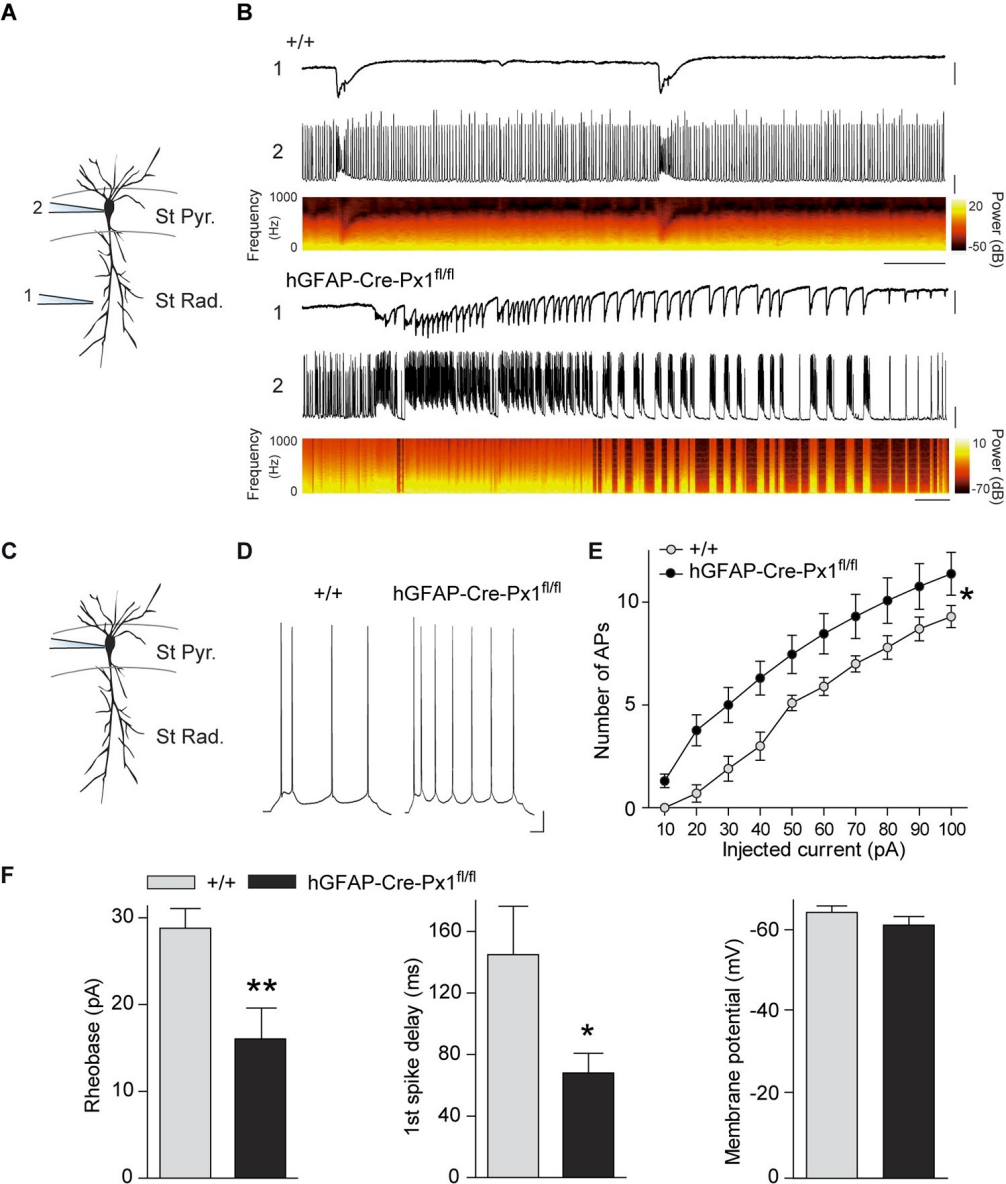
Pyramidal cells from astroglial Px1-deficient mice are more excitable. (**A**) Schematic illustration of simultaneous recording of fEPSP (1) and single neuron (2) in the hippocampus. St Pyr., *Stratum Pyramidale*; St Rad., *Stratum Radiatum*. (**B**) Representative traces of fEPSP (1) and single neuron spontaneous activity (2) in +/+ mice (upper traces) or hGFAP-Cre-Px1^fl/fl^ mice (lower traces). The time-frequency plots corresponding to single neuron recordings are shown under the traces. Scale bar: +/+, 2 s; hGFAP-Cre-Px1^fl/fl^, 5 s; fEPSP, 0.2 mV; single neuron, 20 mV. (**C**) Schematic illustration of single neuron recording in CA1 hippocampal area. (**D**) Representative traces of CA1 pyramidal cell firing pattern from +/+ and hGFAP-Cre-Px1^fl/fl^ mice in basal ACSF containing synaptic blockers (picrotoxin 100 μm, NBQX 10 μM, CPP 10 μM) during depolarisation by 10 pA injection for 500 ms. Cells were held at −60 mV. Scale bar: 50 ms, 10 mV. (**E**) Number of APs evoked by current injection from 10 pA to 100 pA (+/+, *n =* 10 neurons from 5 mice; hGFAP-Cre-Px1^fl/fl^, *n* = 13 neurons from 5 mice; two-way ANOVA and multiple comparisons test). (**F**) Quantification of rheobase, time to first spike and membrane potential, (+/+, *n* = 10 neurons from 5 mice; hGFAP-Cre-Px1^fl/fl^, *n* = 14 neurons from 5 mice; unpaired Student *t* test). Asterisks indicate statistical significance (*p < 0.05, **p < 0.01). The data underlying this figure can be found in the S1 Metadata D tab.

How do astroglial Px1 modulate neuronal network pattern? To examine the contribution of single neurons to the altered network activity in hGFAP-Cre-Px1^fl/fl^ mice, we characterised the electrophysiological properties of CA1 pyramidal cells during bursts and paroxysmal events in control and astroglial Px1-deficient mice by performing simultaneous field potential and patch clamp recordings (Fig 4A). While in +/+ mice, neurons displayed low frequency bursts of activity, as observed extracellularly, and action potentials (AP) firing in between bursts, neurons from hGFAP-Cre-Px1^fl/fl^ mice displayed paroxysmal activity and AP firing between seizures (Fig 4B). These results suggest enhanced hippocampal pyramidal neuron excitability in hGFAP-Cre-Px1^fl/fl^ mice.

We therefore investigated whether astroglial Px1 alters intrinsic membrane properties and excitability of pyramidal cells in basal conditions using whole-cell patch clamp recordings while blocking synaptic activity (Fig 4C). Neuronal excitability was increased, as shown by the enhanced firing rate of pyramidal cells in response to depolarising current pulses (+/+: *n =* 10 neurons from 5 mice, hGFAP-Cre-Px1^fl/fl^: *n* = 13 neurons from 5 mice; Fig 4D and 4E), and by the reduction of both the rheobase (approximately −40%), i.e., the minimal current necessary to evoke an AP, and the delay to first spike (rheobase, +/+: 28.8 ± 2.3 pA, hGFAP-Cre-Px1^fl/fl^: 16 ± 4 pA; *p* = 0.009; first spike delay, +/+: 144.9 ± 31.4 ms, hGFAP-Cre-Px1^fl/fl^: 58.5 ± 14 ms; *p* = 0.025; +/+: *n* = 10 neurons from 5 mice, hGFAP-Cre-Px1^fl/fl^: *n* = 14 neurons from 5 mice; Fig 4F). These changes were not due to alterations in pyramidal cell resting membrane potential and membrane resistance in hGFAP-Cre-Px1^fl/fl^ mice (Vm, +/+: −62.9 ± 2.4 mV, *n* = 10; hGFAP-Cre-Px1^fl/fl^: −59.9 ± 3.8 mV, *n* = 14; Fig 4F; Rm, +/+: 223.1 ± 15.1 MΩ, *n* = 10; hGFAP-Cre-Px1^fl/fl^: 245.6 ± 25.8 MΩ, *n* = 13). We therefore show that astroglial Px1 tunes excitability but not intrinsic properties of pyramidal neurons.

### Astroglial Px1 regulates neuronal network activity and excitability via A1R signalling

**Fig 5:**
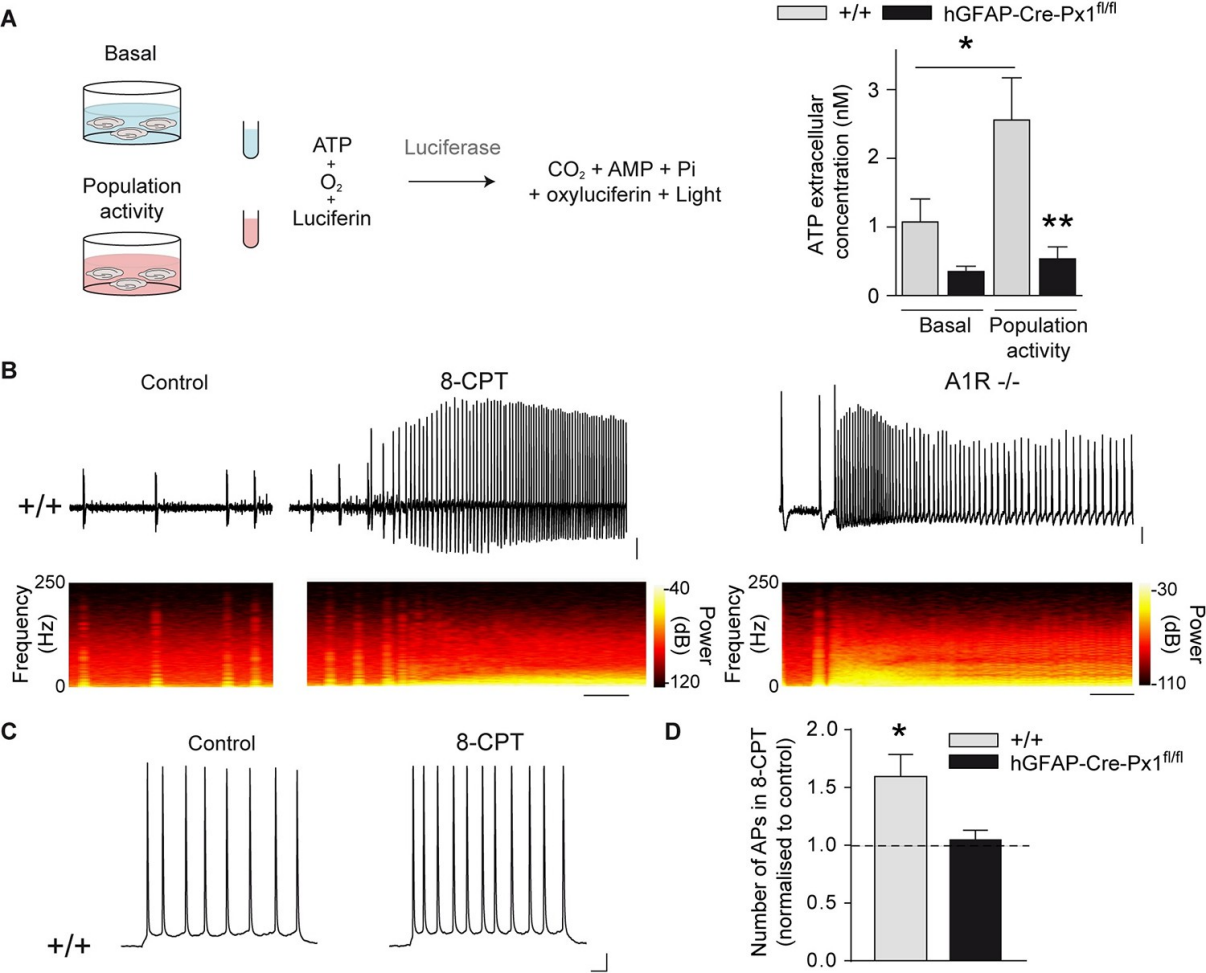
A1R signalling mediates the astroglial Px1 regulation of network activity and excitability. (**A**) Schematic diagram of the experimental design used to measure extracellular ATP concentration (left panel) and quantification of extracellular ATP concentration in 500 μl of ACSF in basal and population activity conditions (+/+: Basal, *n =* 7 mice, Population activity, *n* = 6 mice; hGFAP-Cre-Px1^fl/fl^: Basal, *n* = 8 mice, Population activity, *n* = 8 mice; right panel; one-way ANOVA and Bonferroni post hoc test). (**B**) Representative traces of neuronal network activity in +/+ mice before and during application of the A1R antagonist 8-CPT (1 μM; *n* = 9 slices from 3 mice; scale bar, 20 s, 50 μV) and in A1R^−/−^ mice (*n* = 10 slices from 3 mice; scale bar, 10 s, 100 μV). The corresponding time-frequency plots are shown under the traces. (**C**) Representative traces of CA1 pyramidal cell firing pattern from +/+ and hGFAP-Cre-Px1^fl/fl^ mice in condition of population activity before and after application of 8-CPT during depolarisation by 10 pA injection for 500 ms. Scale bar: 50 ms, 10 mV. (**D**) Number of APs induced by 10 pA current injection, after application of the A1R antagonist 8-CPT, normalised to control (+/+, *n =* 7 neurons from 6 mice; hGFAP-Cre-Px1^fl/fl^, *n* = 7 neurons from 3 mice; Student *t* test). Asterisks indicate statistical significance (*p < 0.05). The data underlying this figure can be found in the S1 Metadata E tab.

**Fig 6:**
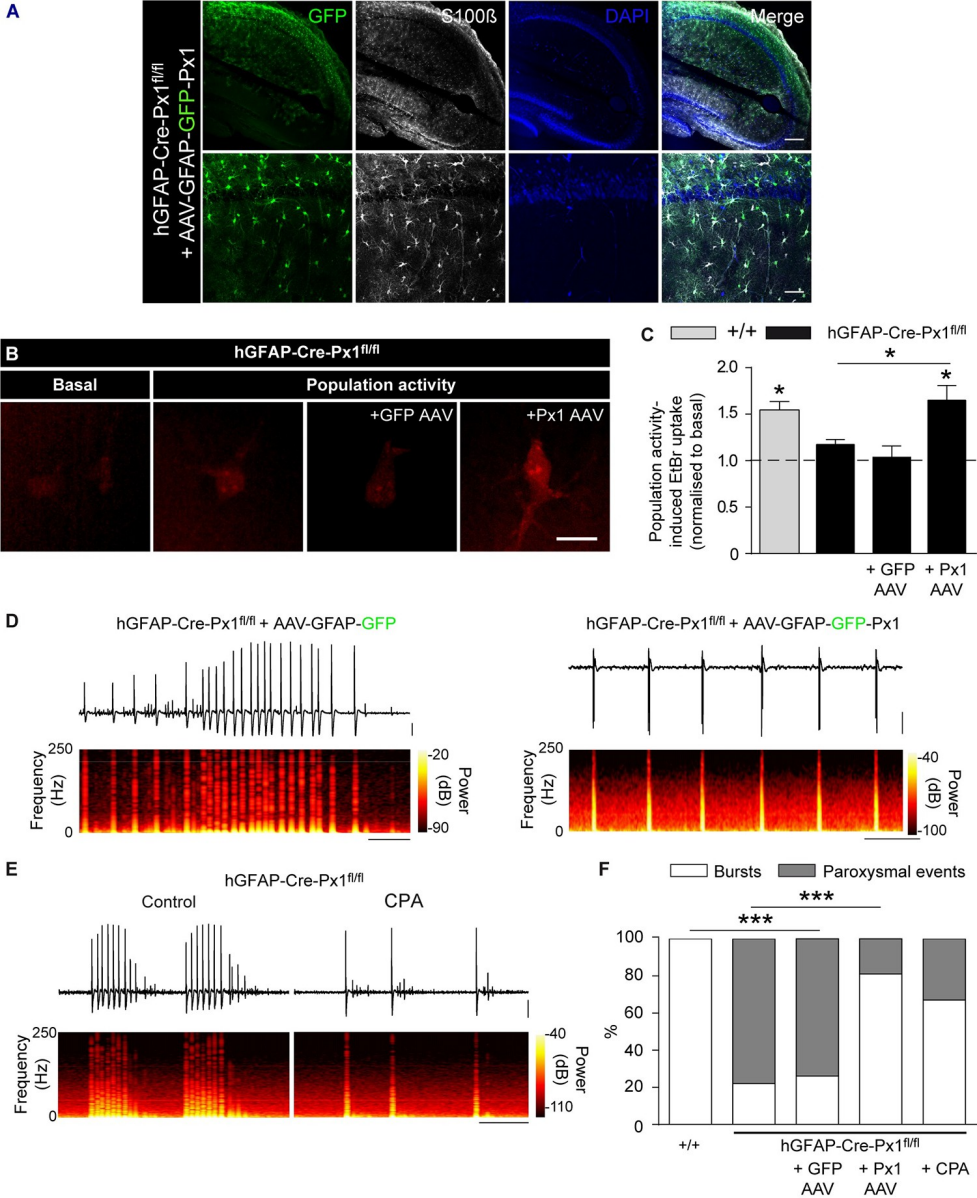
Restoring Px1 expression in astrocytes or pharmacological activation of A1Rs rescue bursting patterns in hGFAP-Cre-Px1^fl/fl^ mice. (**A**) Representative confocal images of GFP (green), S100 (grey), and DAPI (blue) immunolabelling in hippocampal slices from hGFAP-Cre-Px1^fl/fl^ mice infected with AAV-GFAP-GFP-Px1 (Px1 AAV; see Materials and methods). Scale bars, upper panel, 200 μm; lower panel, 50 μm. (**B**) Astroglial EtBr uptake in basal or population activity conditions in hGFAP-Cre-Px1^fl/fl^ astrocytes without or with GFP AAV and Px1 AAV expression. Scale bar, 10 μm. (**C**) Quantification of astroglial activity-dependent EtBr uptake normalised to control conditions in slices from +/+ and hGFAP-Cre-Px1^fl/fl^ mice injected or not with GFP AAV and Px1 AAV (*n =* 5 and 5 mice, respectively; Student *t* test). (**D**) Representative trace of neuronal network activity recorded in hippocampal slices from hGFAP-Cre-Px1^fl/fl^ mice infected with GFP AAV (top; *n* = 19 slices from 4 mice) and Px1 AAV (bottom; *n =* 21 slices from 4 mice). The corresponding time-frequency plots are shown under the traces. Scale bar, 10 s, 100 μV. (**E**) Representative traces of neuronal network activity in hGFAP-Cre-Px1^fl/fl^ mice before and during application of the A1R agonist CPA (300 nM; *n* = 6 slices from 3 mice). The corresponding time-frequency plots are shown under the traces. Scale bar, 25 s, 100 μV. (**F**) Proportion of bursts and paroxysmal events recorded in hippocampal slices from +/+, hGFAP-Cre-Px1^fl/fl^, hGFAP-Cre-Px1^fl/fl^ + GFP AAV, hGFAP-Cre-Px1^fl/fl^ + Px1 AAV, and hGFAP-Cre-Px1^fl/fl^ + CPA mice. Asterisks indicate statistical significance (*p < 0.05, **p < 0.01). The data underlying this figure can be found in the S1 Metadata F tab.

We next further addressed the mechanism implicated in astroglial Px1-dependent modulation of neuronal network activity and single neuron excitability. Astrocytes can regulate neuronal activity via release of neuroactive molecules [25] through various pathways including Px1 channels [9,10]. We therefore hypothesised that Px1 channels, when activated during population activity, release molecules that limit neuronal excitability and prevent paroxysmal events. To test this postulate, we compared extracellular levels of ATP, previously described to be released by Px1 channels [9], during basal and population activity in +/+ and hGFAP-Cre-Px1^fl/fl^ mice, and found that ATP extracellular levels, measured using a luciferin–luciferase assay (Fig 5A, left panel), significantly increased during bursting activity compared to basal conditions in +/+ mice (Basal: 1.07 ± 0.33 nM, Population activity: 2.55 ± 0.61 nM; *n =* 7 mice; *p* = 0.026; Fig 5A, right panel). Notably, slices from hGFAP-Cre-Px1^fl/fl^ mice displayed a marked decrease in ATP extracellular concentration in conditions of sustained activity compared to +/+ mice (0.53 ± 0.17 nM; n = 8 mice; *p* = 0.001; Fig 5A, right panel), suggesting a role for purinergic release in the astroglial Px1-mediated regulation of neuronal network activity. Through which target does Px1-released ATP control neuronal activity? ATP signalling is multifold, in that it leads to both excitation and inhibition of neuronal activity, depending on its targets. ATP is an agonist of P2X and P2Y receptors and can be cleaved by enzymatic hydrolysis to adenosine, which in turn binds A1 and A2 receptors (A1R and A2R) [26]. Further, ATP also modulates the activity of ATP-sensitive potassium channels (K_ATP_) [27]. To examine whether mimicking the strong depletion of ATP measured in slices from hGFAP-Cre-Px1^fl/fl^ mice can induce a switch from bursting activity to paroxysmal activity, we applied ATP or adenosine receptors antagonists in +/+ slices. Antagonists for P2X and P2Y receptors (PPADS and RB2, respectively), K_ATP_ channels (tolbutamide), and A2R (SCH58261) did not induce paroxysmal activity (S6 Fig). However, paroxysmal events were induced by either acutely blocking A1R pharmacologically (8-CPT) in all +/+ slices recorded (*n =* 9 slices from 3 mice; *p* < 0.0001, *n* = 9), or by genetic deletion using A1R −/− mice in 70% of the slices (*n* = 10 slices from 3 mice; Fig 5B and S1 Table), thus mimicking the network activity pattern recorded in hGFAP-Cre-Px1^fl/fl^ mice. Consistently, antagonising A1R in conditions of sustained activity increased excitability of CA1 pyramidal neurons from +/+, but not from hGFAP-Cre-Px1^fl/fl^ mice, as the number of APs elicited by membrane depolarisation in the presence of synaptic blockers was increased (+/+: *n =* 7 neurons from 6 mice, hGFAP-Cre-Px1^fl/fl^: *n* = 7 neurons from 3 mice; *p* = 0.039; Fig 5C and 5D). To confirm the specific role of astroglial Px1 channels and A1Rs in the network inhibition process, we restored in vivo postnatally Px1 expression selectively in hippocampal astrocytes of hGFAP-Cre-Px1^fl/fl^ mice using adeno-associated viral vectors (Fig 6A), or applied the A1R agonist CPA (Fig 6E). We found that restoring Px1 expression in astrocytes from hGFAP-Cre-Px1^fl/fl^ mice recovered activity-dependent EtBr uptake induced by sustained network activity (population activity-induced-EtBr uptake normalised to basal ACSF: astrocytes from hGFAP-Cre-Px1^fl/fl^ mice, 117 ± 5%; astrocytes from hGFAP-Cre-Px1^fl/fl^ mice + AAV Px1, 165 ± 16%, *p* = 0.0243, *n =* 5 mice; Fig 6B and 6C). Conversely, this effect was not observed using a control AAV driving the expression of GFP selectively in astrocytes (GFP AAV; astrocytes from hGFAP-Cre-Px1^fl/fl^ mice + GFP AAV, 103 ± 8%, *p* = 0.9763, *n =* 5 mice; Fig 6B and 6C). In addition, treatment with CPA or postnatal viral expression of Px1 in astrocytes, but not control GFP, rescued a +/+ bursting pattern in hGFAP-Cre-Px1^fl/fl^ mice (Fig 6D–6F). Taken together, our data indicate that astroglial Px1 channels limit neuronal network activity and excitability via A1R signalling.

### Astroglial Px1 restrains neuronal network activity via A1R-mediated regulation of HCN channels

**Fig 7:**
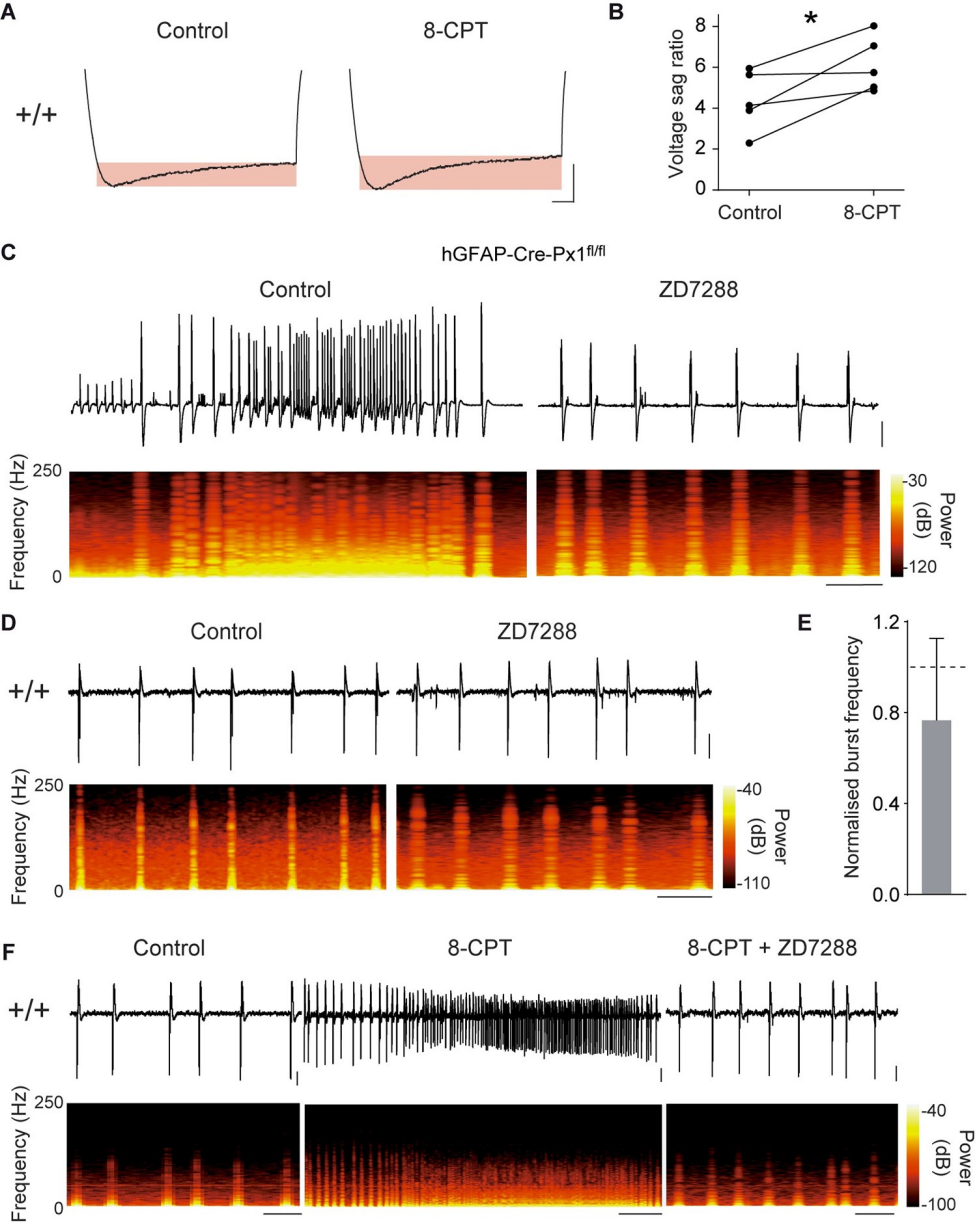
Astrocytic Px1 limits network activity through A1R-mediated modulation of HCN channels. (**A**) Representative traces of the voltage sag ratio in +/+ mice before and after application of the A1R antagonist 8-CPT. Scale bar: 50 ms, 10 mV. (**B**) Quantification of voltage sag ratio, defined as ((Vmin − Vend)/Vmin) × 100 (*n* = 5 neurons from 4 mice, paired Student *t* test). (**C-D**) Representative traces of neuronal network activity recorded in hGFAP-Cre-Px1^fl/fl^ mice (**C**) and +/+ mice (**D**) before and during application of the HCN channel antagonist ZD7288 (10 μM; hGFAP-Cre-Px1^fl/fl^, *n* = 5 slices from 4 mice; +/+, *n* = 6 slices from 2 mice). The corresponding time-frequency plots are shown under the traces. Scale bars, in c: 10 s, 200 μV; in d: 10 s, 50 μV. (**E**) Quantification of the change in burst frequency in +/+ mice after application of ZD7288 (paired Student *t* test). (**F**) Representative traces of neuronal network activity recorded in the same hippocampal slice before and after subsequent applications of 8-CPT and ZD7288 in +/+ mice (*n* = 5 slices from 4 mice). The corresponding time-frequency plots are shown under the traces. Scale bars, control and 8-CPT + ZD7288: 10 s, 25 μV; 8-CPT: 25 s, 25 μV. Asterisks indicate statistical significance (*p < 0.05). The data underlying this figure can be found in the S1 Metadata G tab.

Several A1R-dependent mechanisms have been described to impact neuronal excitability. G protein-coupled inwardly rectifying potassium channels (GIRK) activation by A1R-dependent intracellular processes was reported to decrease neuronal excitability [28]. We thus investigated whether inhibiting GIRK channels in +/+ mice can mimick the paroxysmal activity observed in hGFAP-Cre-Px1^fl/fl^ mice. However, we found that GIRK inhibition by SCH23390 failed to induce paroxystic activity, and only increased the frequency of bursts (*n =* 5; *p* = 0.005; S6 Fig). Alternatively, A1R-mediated partial inhibition of HCN-gated channels has been reported to decrease neuronal excitability [29,30]. To determine whether HCN channels are indeed endogenously inhibited by A1R signalling during sustained activity in +/+ mice, we measured the voltage sag ratio, which reflects the I_h_ current mediated by HCN channels activation, using whole-cell patch clamp recording of pyramidal cells. We found that blockade of A1R with 8-CPT indeed increased the voltage sag ratio in +/+ mice (+/+: *n =* 5 neurons from 4 mice; *p* = 0.039; Fig 7A and 7B).

**Fig 8:**
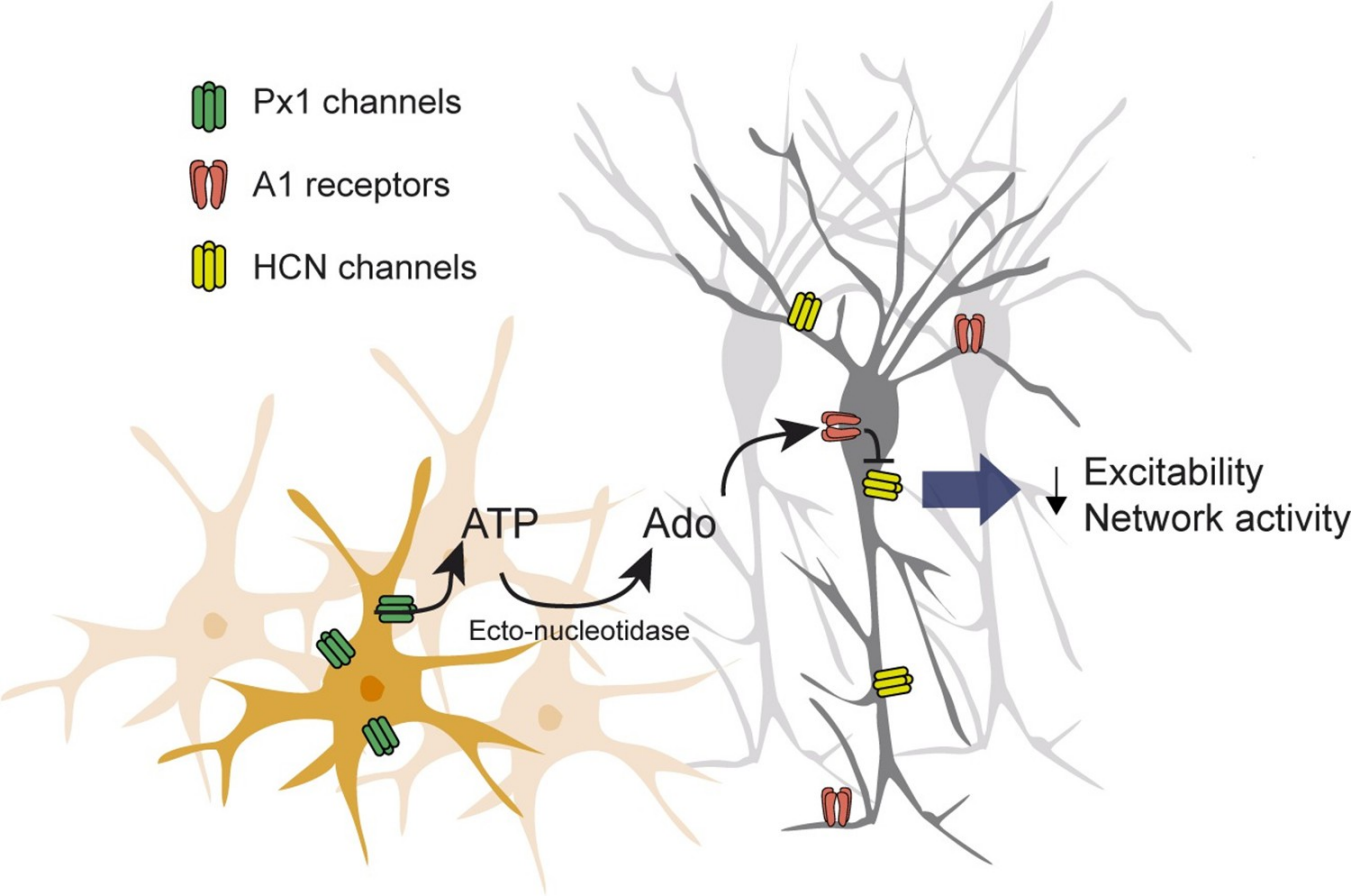
Mechanism of Px1-mediated modulation of network activity. Schematic diagram depicting the proposed mechanism through which astroglial Px1 channels signalling decreases population activity: Px1 channels release ATP, converted to adenosine, which binds neuronal A1 receptors. Subsequent intracellular signalling induces inhibition of HCN channels, leading to decreased excitability and population activity. Ado, Adenosine.

Does this pathway set neuronal network pattern? To evaluate whether blockade of HCN channels can restore a bursting phenotype in hGFAP-Cre-Px1^fl/fl^ mice, we inhibited HCN channels with ZD7288 and indeed found that this switched the activity pattern from paroxysmal to bursting activity (*n =* 5 slices from 4 mice; Fig 7C), while it had no effect on the bursting pattern in +/+ mice (*n* = 5 slices from 4 mice; *p* = 0.513 and *p* = 0.486 for burst frequency and duration, respectively; Fig 7D and 7E and S1 Table). Interestingly, the bursting pattern induced by ZD7288 in hGFAP-Cre-Px1^fl/fl^ mice (*n =* 5 slices from 4 mice) did not differ from that observed in +/+ mice (*n* = 6 slices from 2 mice; *p* = 0.339 and 0.441 for burst frequency and duration, respectively; S1 Table). Lastly, to ensure that the A1R-mediated negative control of HCN channels can limit population activity, we induced paroxysmal activity in all tested hippocampal slices from +/+ mice by inhibiting A1Rs with 8-CPT (*n =* 5 slices from 4 mice, *p* < 0.0001) and subsequently blocked HCN channels with ZD7288. Consistently, HCN channel antagonism inhibited paroxysmal events caused by A1R blockade, systematically reverting the electrophysiological phenotype to a bursting pattern (*n =* 5 slices from 4 mice, *p* < 0.0001), which was similar to the one displayed in control condition (Fig 7F; *p* = 0.3 and *p* = 0.189 for burst frequency and duration, respectively, *n* = 5 slices from 4 mice). Altogether, these data suggest that Px1 channels in astrocytes tune down population activity through purinergic signalling-mediated regulation of HCN channels (Fig 8).

## Discussion

The present findings establish astroglial Px1 channels as major contributors to the regulation of hippocampal neuronal network patterns. Activation of astroglial Px1 channels by sustained neuronal activity inhibits population activity through purinergic signalling. Indeed, we show that Px1 channels release ATP and that activation of A1 receptors by its metabolite adenosine inhibits HCN channels, leading to decreased neuronal excitability and hippocampal network activity.

### A role for astroglial Px1 channels in neuronal network activity

We here show for the first time Px1 mRNA expression in hippocampal astrocytes from 10 days postnatally. Px1 mRNA expression persisted throughout development, with a significant increase in adult (P50), compared to juvenile (P10) mice. While Px1 protein expression has been inferred in astrocyte cultures from the presence of outward currents blocked by mefloquine [22] and reported in brain slices [16], Px1 mRNA expression was only measured irrespective of cell type and was surprisingly shown to decrease during development [7]. This suggests a potential differential expression regulation of astroglial and neuronal Px1.

Further dissection of Px1 channel functional properties revealed that Px1 channels opening in both neurons and astrocytes was triggered by sustained network activity. Inhibition of EtBr uptake by blockade with the peptide ^10^Panx ascertained the specific implication of Px1 in the activity-dependent astroglial channel opening, which is consistent with reports of enhanced Px1 channel function as a response to sustained neuronal activity and pathological insult such as kainate-induced seizures [16] or acute stress [23]. This activity-dependent Px1 function may result from changes in channel conformation conferring distinct biophysical properties [31]. Px1 channels can indeed display a high-conductance state of approximately 500 pS, associated with ATP permeability, in physiological or pathological conditions characterised by increased extracellular K^+^ levels [32]; and a low-conductance state of approximately 50 pS, with no permeability to ATP, which is induced by voltage changes in the absence of K^+^ and whose physiological function is yet unknown [31].

### Astroglial Px1 channels inhibit hippocampal neuronal networks

Strikingly, astroglial Px1 deficiency induced a switch from bursting pattern to paroxysmal activity, indicating an inhibitory role of astroglial Px1 channels in hippocampal neuronal networks. Corroborating this observation, astroglial Px1 deficiency increased single neuron excitability. Indeed, neurons from glial conditional Px1 knockout mice exhibited normal resting membrane potentials but fired a larger number of APs with a shorter delay upon depolarisation. These findings indicate that astroglial Px1 channel do not set excitability through modulation of neuronal basal membrane potential.

Contrasting roles for Px1 channels have emerged from reports concerning their function in presence of a regime of intense neuronal activity: on one hand, Px1 channel inhibition or KO was proposed to decrease bursts frequency and amplitude ex vivo [14], to ameliorate seizure outcome in vivo [16] and to suppress seizures in tissue resected from epileptic patients [33]. On the other hand, Px1-mediated signalling was observed to decrease neuronal excitability [20] and muscarinic acetylcholine-mediated seizure susceptibility [15]. It is, however, crucial to note that the tools used to target Px1 channels in the aforementioned studies did not permit to discriminate between cell types. We here show that full deletion of Px1 fails to induce paroxysmal activity, suggesting that neuronal Px1 function may be distinct from that of astroglial Px1. Specific functions of Px1 might arise from cell type-dependent opening mechanisms: Px1 opening was shown to be triggered by NMDA receptor, P2Y and P2X7 receptor activation, as well as intracellular calcium signals [6,14,15], all of which may be cell type-specific. Alternatively, upon activation, Px1 channels downstream signalling might vary.

ATP released via Px1 channels and its metabolites can theoretically have several targets in different cell types. In population activity regime, we found that HCN channels are the targets of the astroglial Px1 pathway we identified. One possibility is that the spatial proximity of astroglial ATP release site to other cell compartments, as well as the local density and activity of ecto-nucleotidase metabolising ATP into adenosine in the vicinity of the release site may determine their preferential modulation in this regime of activity.

### Astroglial Px1 channels regulate neuronal excitability through A1 receptors and HCN channels

Px1 channels nonselectively conduit small molecules. We show that intense neuronal activity triggers ATP release from astroglial Px1 channels. Further examination of the downstream effect of this ATP excess unveiled a prominent role for adenosine as an inhibitory signal, as antagonising and activating A1R respectively mimicked and abolished the paroxysmal activity recorded in astroglial Px1-deficient mice. In accordance with these data, two studies have identified Px1 and A1R as part of a common signalling pathway affecting sleep–wake cycle [34] and neuronal metabolism [20], whereby Px1 channels release ATP, and its metabolite adenosine then activates A1 receptors to regulate neuronal activity.

A1R activation impact on neuronal excitability is, to date, sparsely documented. Available reports suggest that its inhibitory action involves the modulation of GIRK and HCN channels [20,28–30]. Under sustained neuronal activity, we observed an implication of HCN channels, of which the inhibition suppressed both astroglial Px1 deficiency and A1R antagonism-mediated paroxysmal activity. It has been suggested that A1R activation inhibits adenylyl cyclase activity and thereby cAMP production and HCN activation, decreasing cell excitability [35]. In the present study, we demonstrate that astroglial Px1 channels inhibit hippocampal neuronal networks through a mechanism involving purinergic A1R and HCN signalling under sustained neuronal activity. The activity-dependence of such process is suggestive of a mechanism engaged during synchronous sustained neuronal discharges. The therapeutic potential of targeting Px1 should, however, take into consideration the apparent cell type-differential effect of its inhibition.

## Materials and methods

### Ethics statement

Experiments were performed according to the guidelines of European Community Council Directives of January 1, 2013 (2010/63/EU), and of the local animal welfare committee (certificate A751901, Ministere de l’Agriculture et de l’Alimentation) and were approved by our local ethics committee and by the ministry of education and research (n° 2015071010466740).

### Animals

Mice (*Mus musculus*) were group housed on a 12-h light/dark cycle. All efforts were made to minimise the number of animals used and their suffering. Experiments were performed on the hippocampus of wild-type C57BL/6 mice (+/+), which were bred in the laboratory, hGFAP-Cre-Px1^fl/fl^ mice, hGFAP-CreERT2-Px1^fl/fl^ mice, constitutive Px1^−/−^ mice, ADORA (A1R^−/^) mice, obtained from Jackson, Aldhl1:L10a-eGFP mice, obtained from the laboratory of N. Heintz (Rockefeller University, NY, USA) and hGFAP-creERT2-*mT/mG* mice, obtained from the laboratory of P. Billuart [36] by crossing hGFAP-creERT2 mice with *mT/mG* mice (Jackson strain# 007576). hGFAP-Cre-Px1^fl/fl^ and hGFAP-CreERT2-Px1^fl/fl^ were generated in the laboratory and present conditional constitutive or inducible (after TF treatment) deletion of Px1 in GFAP-expressing cells, respectively. Global constitutive Px1^−/−^ mice were generated in the laboratory and present ubiquitous Px1 deletion. All mice had a C57BL/6 background and were housed in the same conditions. To control for germline recombination in hGFAP-Cre-Px1^fl/fl^, GFAP-CreERT2-Px1^fl/fl^ and constitutive Px1^−/−^ mice, breedings were performed with Cre-bearing males and Cre-negative females.

For all analyses, mice of both genders were used at postnatal days 16 to 25 for ex vivo electrophysiology, as previously described [2], while 6-week-old male were used for in vivo EEG recordings.

### hGFAP-Cre-Px1^fl/fl^, hGFAP-CreERT2-Px1^fl/fl^, and constitutive Px1^−/−^mice generation

To generate the Px1 knockout mouse lines, five strains of mice were used: a recombinant Panx1 KOMP (Panx1^tm1a(KOMP)Wtsi^) strain [37], and four deleter mouse strains, the hGFAP-Cre, PGK-Cre KI mice, the Actb-Flpe transgenic mice [38], and the hGFAP-CreERT2 deleter mice (obtained from the laboratory of F. Kirchhoff; [39]).

Briefly, the Panx1 tm1a allele integrates in intron 2 an artificial exon containing a reporter cassette (IRES-LacZ) that creates a transcriptional stop after exon2 with concomitant LacZ expression, and a second cassette (Neo) for selection purposes (S2A Fig). These two cassettes are surrounded by FRT sites that allow the excision of all the cassettes by Flp recombinase. This is obtained by breeding Panx1^tm1a(KOMP)Wtsi^ mice with deleter ActB-Flpe mice, leading to mice bearing the conditional allele Panx1^tm1c(KOMP)Wtsi^ (S2A Fig).

To constitutively knockout Px1 in all cell types, the Panx1^tm1c(KOMP)Wtsi^ mice were crossed with the PGK-Cre deleter mice, which express the Cre-recombinase under the control of the PGK promoter. This results in Px1 exon 3 excision in all expressing cells (data shown in S5 Fig).

To conditionnally knockout Px1 in astrocytes, the Panx1^tm1c(KOMP)Wtsi^ mice were crossed with the hGFAP-Cre deleter mice, which express the Cre-recombinase under the control of the GFAP promoter. This results in Px1 exon 3 excision in GFAP-expressing cells (S2A Fig).

To conditionally and inducibly knockout Px1 in astrocytes, the Panx1^tm1c(KOMP)Wtsi^ mice were crossed with the hGFAP-CreERT2 deleter mice, expressing the CreERT2-recombinase under the control of the GFAP promoter (data shown in S4 Fig).

The Panx1^tm1a(KOMP)Wtsi^ was generated directly on C57BL/6J genetic background. The Cre and Flpe deleter strains have been backcrossed more than 10 generations and maintained on C57BL/6J genetic background.

### Tamoxifen-induced DNA recombination

To induce DNA recombination in hGFAP-CreERT2-Px1^fl/fl^ and in GFAPcreERT2-*mT/mG* mice, TF (10 mg/ml in corn oil, Sigma) was IP injected into P15 mice for 5 consecutive days (100 mg/kg body weight). This results in Px1 exon 3 excision (hGFAP-CreERT2-Px1^fl/fl^ mice) and in GFP expression (GFAPcreERT2-*mT/mG* mice) in GFAP-expressing cells. Two weeks after injection, hGFAP-CreERT2-Px1^fl/fl^ mice were used for FISH and MEA recordings, and GFAPcreERT2-*mT/mG* mice for immunohistochemistry (data shown in S4 Fig).

### Translating Ribosome Affinity Purification (TRAP) in astrocytes and quantitative RT-PCR

Astroglial polysomal RNAs were extracted from postnatal days 10 to 50 Aldh1l1:L10a-eGFP mice hippocampi by using the Translating Ribosome Affinity Purification (TRAP; [40]) followed by extraction with RNeasy Lipid tissue kit (Qiagen, USA). cDNAs were synthetised from 1 μg RNA using Reverse Transcriptase Superscript III (Thermo Fisher, USA) and stored at −80°C. PCR was performed in triplicate on a LC480 Roche Light cycler on 1 μl cDNA using SybrGreen master mix (Roche, Switzerland). The cycle used was 50°C for 2 min, 95°C for 5 min, and 40 cycles of 95°C for 15 s and 60°C for 1 min. The relative abundance of amplified cDNA was calculated as 2^−ΔCt^, where ΔCt (change in cycle threshold) equals Ct in P30 and P50 samples minus Ct in P10 samples. Results are expressed as means of 2^−ΔCt^ tested cDNA/ 2^−ΔCt^ RNA18s values. Experiments were done on three independent pools of hippocampi of three mice each. The following primers were used: Px1 forward 5′-CCTGCAGAGCGAGTCTGGAA-3′; Px1 reverse 5′-TGCGGGCAGGTACAGGAGTA-3′; RNA18s forward 5′-TTGAAAATCCGGGGGAGAG-3′; RNA 18s reverse 5′-ACATTGTTCCAACATGCCAG-3′.

### High-resolution fluorescent in situ hybridisation (FISH) by RNAscope

FISH was performed on frozen brain sections from mice perfused with 4% paraformaldehyde, following the RNAscope procedures (Advanced Cell Diagnostics, Newark, Ca, USA). Hybridisation of probe against *Bacillus subtilis* dihydrodipicolinate reductase (dapB) gene was used as a negative control. A probe against exon 3 of Px1 (744–1000, 019482) was used. Labelled cells from the hippocampus were examined with a spinning disk confocal microscope (EclipseTi, Nikon) equipped with CMOS camera (Photometrics). Stacks of consecutive images (5 stacks/brain section; 3 brain sections/mouse) with high-bit depth colour (16 bit) with a pixel size of 0.108 μm were taken with a 60× objective at 1 μm intervals and acquired sequentially with 4 lasers (405, 488, 561, and 647 nm). Astrocyte-specific FISH dots were recognized based on their colocalisation with S100β immunolabelling, using the AstroDot ImageJ plug-in as previously described [41,42]. Single plane images are illustrated to show localisation of Px1 probe in neurons and astrocytes.

### Acute hippocampal slice preparation

Acute transverse hippocampal slices (300 to 400 μm) from P16-P25 mice were prepared as previously described [2]. Briefly, slices were cut at low speed (0.04 mm/s) and at a vibration frequency of 70 Hz in ice-cold oxygenated ACSF supplemented with sucrose (in mM: 87 NaCl, 2.5 KCl, 2.5 CaCl_2_, 7 MgCl, 1 NaH_2_PO_4_, 25 NaHCO_3_, and 10 glucose, saturated with 95% O_2_ and 5% CO_2_). Slices were maintained in a storage chamber containing standard ACSF (in mM: 119 NaCl, 2.5 KCl, 2.5 CaCl_2_, 1.3 MgSO_4_, 1 NaH_2_PO_4_, 26.2 NaHCO_3_, and 11 glucose, saturated with 95% O_2_ and 5% CO_2_) for 30 min, and then stored for at least 1 hour before recording in a pro-bursting (0 mM Mg^2+^, 6 mM K^+^) ACSF.

### Ex vivo electrophysiology

Multielectrode array (MEA), field excitatory postsynaptic potential (fEPSP), and whole-cell patch clamp recordings were performed. Hippocampal slices were transferred on planar MEA petri dishes (200–30 ITO electrodes organised in a 12 × 12 matrix, with internal reference, 30 μm diameter and 200 μm interelectrode distance; Multichannel Systems, Germany) and kept in place using a small platinum anchor. The slices were continuously perfused at a rate of 2 ml/min with ACSF for bursting and paroxystic activity recordings. Pictures of hippocampal slices on MEAs were acquired with a video microscope table (MEA-VMT1; Multichannel Systems, Germany) through MEA Monitor software (Multichannel Systems, Germany) to identify the location of the electrodes on the hippocampus and to select electrodes of interest. Data were sampled at 10 kHz and network spontaneous activity was recorded at room temperature by MEA2100-120 system (bandwidth 1 to 3,000 Hz, gain 5×, Multichannel Systems, Germany) through the MC Rack 4.5.1 software (Multichannel Systems, Germany). fEPSPs were recorded with glass pipettes (2 to 5 MΩ) filled with ACSF and placed in the *stratum radiatum*. For whole-cell patch clamp recordings of CA1 pyramidal neurons, slices were transferred to a submerged recording chamber mounted on a S-SCOPE II microscope (Scientifica, UK) equipped with infrared-differential interference microscopy and were perfused with standard or pro-bursting ACSF at a rate of 2 ml/min. Whole-cell recordings were obtained from visually identified CA1 pyramidal neurons and astrocytes using glass pipettes (4 to 7 MΩ) filled with (in mM): 105 K-gluconate, 30 KCl, 10 HEPES, 10 phosphocreatine, 4 Mg–adenosine triphosphate (ATP), 0.3 guanosine triphosphate (GTP)-tris, and 0.3 EGTA (pH 7.4, 280 mOsmol). Membrane potentials of neurons recorded from both groups were clamped at −60 mV to allow comparison between groups, except for the evaluation of neuronal resting membrane potentials. Neuronal excitability was assessed in the presence of blockers of inhibitory (GABA_A_R, picrotoxin) and excitatory (AMPAR, NBQX; NMDAR, CPP) synaptic activity. Measurements of membrane resistance and capacitance were performed on pyramidal cells clamped at −60 mV. Patch clamp recordings were acquired with Multiclamp 700B (Axon Instruments, USA), digitised at 10 kHz, filtered at 2 kHz, and analysed using Clampfit 10.2 software (Molecular Devices, USA).

### MEA data analysis

Bursting and paroxysmal activity raw data were analysed with MC Rack (Multichannel Systems, Germany). Detection of bursts was performed using the “Spike Sorter” algorithm, which sets a threshold based on multiples of standard deviation of the noise (5-fold) calculated over the first 500 ms of recording free of electrical activity. A 5-fold standard deviation threshold was used to automatically detect each event, which could be modified in real time by the operator on visual check if needed. Bursts were arbitrarily defined as discharges shorter than 5 s in duration. Typically, bursts were characterised by fast voltage oscillations followed by slow oscillations or negative shifts. To analyse seizure-like activity, data were exported to Neuroexplorer (Nex Technologies, USA). Paroxysmal events were identified as discharges lasting more than 5 s; successive paroxysmal discharges were considered separate events based on their waveform and on the presence of a minimum (>10 s) interval of silent or bursting activity between them [43].

### Time-frequency spectrograms

Time-frequency plots were computed using Neuroexplorer (version 4.109, Nex Technologies, USA), setting the maximum frequency at 1,000 Hz and a time shift of 5 ms for single neuron recordings and 50 ms for MEA recordings. Values were normalised to the logarithm of the power spectral density (PSD) and expressed as dB. After the calculation the spectrum was smoothed with a Gaussian filter. For MEA recordings, only frequencies between 1 and 250 Hz were displayed.

### Power spectral density

PSD was computed using Neuroexplorer (version 4.109, Nex Technologies, USA) setting the maximum frequency at 500 Hz and number of frequency values at 2,048. The PSD computed for each slice was normalised to the percent of the total PSD to allow comparison between slices and no smoothing was used. Data were then binned in frequency windows corresponding to different brain rhythms: delta (1 to 4 Hz), theta (4 to 8 Hz), alpha (8 to 13 Hz), beta (13 to 30 Hz), gamma (30 to 80 Hz), fast (80 to 200 Hz), and oscillations >200 Hz.

### Pilocarpine seizure model and telemetric EEG recording

Six-week-old mice implanted with wireless ETA-F10 DSI EEG electrodes were injected IP with 300 mg/kg of pilocarpine and placed in an open arena for video and EEG recording of the *status epilepticus*. Latency to ictus as well as the survival rate were evaluated. EEG experiments were carried out using wireless ETA-F10 transmitters (Data Sciences International) for EEG recording and video monitoring as previously described [44]. After anaesthesia (ketamine, 95 mg/kg; xylazine, 10 mg/kg; IP), a 1-cm midline sagittal incision was made starting above the skull midline and extending along the neck to create a pocket for subcutaneous placement of the transmitter along the dorsal flank of the animal. The flexible recording electrodes were implanted subdurally through small holes drilled in the skull (stereotaxic coordinates: antero-posterior: −2 mm, medio-lateral: +1.5/−1.5 mm and held in place with dental cement. Mice were allowed to recover for 7 days before recording. EEG signal was collected through DSI radiofrequency receivers placed under each cage. EEG data were acquired at a sampling rate of 200 Hz using the DSI Dataquest A.R.T. system, version 4.33.

### Ethidium bromide uptake assay

To investigate Px1 channel activation, EtBr uptake experiments were performed, as previously described [33,45]. Hippocampal slices were distributed in small customised submerged chambers and incubated in pro-bursting ACSF containing ^10^Panx (400 μM), a Px1 channel specific blocking peptide, or a scramble peptide ^Sc^Panx (400 μM) 15 min before and during the application of ethidium bromide (EtBr; 314Da, 4 μM). EtBr is a hemichannel-permeable sensitive fluorescent tracer that intercalates into DNA strands and has thus the advantage of being sequestered into the nucleus once taken up by cells. Slices were then rinsed for 15 min in ACSF, fixed for 2 h in 4% paraformaldehyde, and immunostained for glial fibrillary acidic protein (GFAP, 1:500; rabbit anti-GFAP antibody, Sigma-Aldrich, France) or S100β (1:500; rabbit anti-S100β antibody, 1:500, Sigma-Aldrich, France), and NeuN (1:500; mouse anti-NeuN antibody, Millipore, France) with goat anti-rabbit and goat anti-mouse secondary antibodies conjugated to Alexa Fluor 488 and 633, respectively (1:1,000; Life Technology, France). After mounting with Fluoromount-G (Southern Biotechnology, USA), labelled cells were examined with a confocal laser-scanning microscope (TCS SP5, Leica, Germany) using a 63× objective. Stacks of consecutive confocal images with high-bit depth colour (16 bit) were taken at 1 μm intervals in sequential mode with lasers (488 nm for S100β and GFAP, 647 nm for NeuN, and 561 nm for EtBr). Cells positive for S100β or GFAP and NeuN were used for dye uptake analysis in astrocytes and neurons, respectively. EtBr fluorescence intensity in astrocytes and neurons was digitised in arbitrary units ranging from 0 to 65,000 (shades of gray of the 16-bit image). EtBr uptake was determined by measuring the difference in fluorescence intensity between the cells and the background in the same field, where no labelled cells were detected, using a homemade plug-in developed for ImageJ/Fiji software [46,47] thanks to Bio-Format [48] and mcib3D [49] libraries. More precisely, the NeuN layer was first identified by applying a Z-projection (standard deviation method), followed by a Gaussian blur (size = 15) and thresholding of NeuN channel. EtBr fluorescence in neurons was then calculated in the identified NeuN layer. For S100β/GFAP-labelled astrocytes, their nucleus position was first manually defined using the cell counter Fiji plugin and saved as an xml file. Then, the astrocytic nucleus population was detected using the remove outlier plugin (radius = 15, threshold = 1, which = bright), followed by a difference of Gaussians (σ1 = 15, σ2 = 10) and a moments threshold method. Only astrocytic nuclei sharing positions in xml file were kept for analysis. Before labelling the astrocytic nucleus objects, the NeuN layer ROI was filled with zero in binary image. For each astrocytic nucleus volume, EtBr fluorescence intensity was calculated. For each experiment, a basal control condition is included to which all the other tested conditions within the same experiment are compared. For each condition, 3 slices are used; for each slice, 3 image stacks are taken and used to calculate the average fluorescence/slice. Average values from all the slices in the same condition are used to calculate the mean fluorescence value for that same condition. Within an experiment, the effects of the tested conditions are thus always normalised to this internal control condition.

### ATP concentration measurements

ATP release was measured from hippocampal slices maintained in small, customised, submerged chambers containing oxygenated ACSF (3 slices/well with 500 μL of ACSF), as previously described [33,45]. Extracellular ATP levels were determined by using the luciferin–luciferase assay (ATPLite kit, PerkinElmer, USA) and a luminometer (Tristar, Berthold). Each condition was run in quadruplicate, and absolute values were obtained from ATP standards.

### Adeno-associated viral infections

For AAV in vivo gene transfer, a transgene composed of GFP or GFP-Px1 cDNA separated by a P2A sequence in a single open reading frame was placed under the control of a GFAP-specific promoter in an AAV shuttle plasmid containing the inverted terminal repeats (ITRs) of AAV2 (AAV-GFAP-GFP and AAV-GFAP-GFP-Px1, respectively). Pseudotyped serotype 9 AAV particles were produced by transient cotransfection of HEK-293T cells, as previously described [50]. Viral titres were determined by qPCR amplification of the ITR on DNase-resistant particles and expressed as vector genome per ml (vg/ml). P15 *−/−* mice were anesthetised with a mixture of ketamine (95 mg/kg; Merial) and xylazine (10 mg/kg; Bayer) in 0.9% NaCl and placed on a stereotaxic frame with constant body temperature monitoring. AAVs were diluted in PBS with 1% BSA at a concentration of 1.46 × 10^13^ vg/ml, and 1 μl of virus was stereotaxically injected unilaterally into the hippocampi at a rate of 0.1 μl/min, using a 29-gauge blunt-tip needle linked to a 2-μl Hamilton syringe (Phymep). The stereotaxic coordinates to Bregma were: antero-posterior, −1.94 mm; medio-lateral: ±1.5 mm; and dorso-ventral, −1.35 mm. After the injection, the needle was left in place for 5 min before being slowly removed. The skin was glued, and mice recovery was checked for the next 24 h. After 2 to 4 weeks, the mice were killed and processed for electrophysiology and dye uptake experiments.

### Drugs

^10^Panx (WRQAAFVDSY) and ^sc^Panx (FSVYWAQADR) were synthesised by Thermo Fisher Scientific (purity, >95%); pyridoxalphosphate-6-azophenyl-2′,4′-disulfonic acid (PPADS), N6-Cyclopentyladenosine (CPA), ZD7288, SCH58261, NBQX, and CPP were purchased from Tocris, Reactive Blue-2 (RB-2), Tolbutamide, SCH23390, 8-Cyclopentyl-1,3-dimethylxanthine (8-CPT), picrotoxin from Sigma-Aldrich and EtBr from Molecular Probes.

### Statistical analysis

Data are expressed as means ± SEM and *n* represents the number of independent experiments. To control for bias, the protocols used in the study were applied rigorously in the same way for all the mouse lines, and for most experiments, a control and a transgenic mouse have been used in parallel on the same day. Furthermore, different experimenters performed experiments and analysis on all the mouse lines. Statistical significance was determined by paired and unpaired *t* tests for between-group comparisons, and by one-way and two-way ANOVAs with Bonferroni or Dunnett post hoc tests for within-group comparisons, as well as by Fisher exact test for distribution comparison. Nonparametric tests were used for discrete variables. Differences were considered significant at *p* < 0.05. Statistical analysis was performed using GraphPad Prism 5 software.

## Supporting information

S1 Fig Pharmacological Px1 inhibition or molecular Px1 disruption decrease astroglial currents at positive potentials. (**A**) Representative astroglial whole-cell current profiles evoked by 150 ms voltage steps (−110 to +100 mV; scale bar, 2 pA, 25 ms) and quantification of current–voltage (I/V) plots and intrinsic membrane properties (**B**) of astrocytes in +/+ mice before (black trace) and after (red trace) inhibition of Px1 channels with the ^10^Panx1 peptide (400 μM, 40 min) (*n =* 4 cells from 3 mice) and in hGFAP-Cre-Px1^fl/fl^ mice (grey trace; *n* = 5 cells from 3 mice). Asterisks indicate statistical significance (two-way repeated measures ANOVA (A) or Student paired *t* test (B), ***p < 0.001, **p < 0.01). The data underlying this figure can be found in the S1 Metadata H tab.
(PDF)

S2 Fig Astroglial Px1 expression and function are disrupted in hGFAP-CrePx1^fl/fl^ mouse. (**A**) Generation of the hGFAP-Cre-Px1^fl/fl^ mouse (see Materials and methods). (**B**) Left, representative confocal images of Px1 mRNA in hippocampus by FISH in the hGFAP-Cre-Px1^fl/fl^ mouse. Neuron nuclei are immunolabelled with NeuN (top images) and astrocytes with S100β (bottom images). Scale bar, 10 μm. Right, quantification of Px1 mRNA (FISH dot density: dots/mm^2^) in neurons and astrocytes in +/+ and hGFAP-Cre-Px1^fl/fl^ mice (*n =* 3 and 3 mice, respectively; Student *t* test). (**C**) EtBr uptake in basal and population activity conditions in +/+ and hGFAP-Cre-Px1^fl/fl^ mice. Scale bar, 20 μm. Insets: zoom on stratum radiatum astrocytes highlighted by a dotted rectangle. Scale bar, 10 μm. s.p., *stratum pyramidale*; s.r., *stratum radiatum*. (**D**) Quantification of neuronal and astroglial EtBr uptake normalised to basal condition in slices from +/+ mice (*n =* 8 mice) and hGFAP-Cre-Px1^fl/fl^ mice treated or not with ^10^Panx (*n* = 14 mice) and ^sc^Panx (*n* = 8 mice; repeated measures one-way ANOVA). Asterisks indicate statistical significance (*p < 0.05, **p < 0.01). The data underlying this figure can be found in the S1 Metadata I tab.
(PDF)

S3 Fig Astroglial Px1-deleted mice display no gross anatomical alterations and normal hippocampal architecture. (**A**) Immunolabelling of neurons (NeuN, red), astrocytes (GFAP, grey), and nuclei (TO-PRO, blue) in +/+ and hGFAP-Cre-Px1^fl/fl^ mice. Scale bar, 200 μm. (**B-C**) Quantification of hippocampal neuron and astrocyte cell density (**B**) and dentate gyrus (DG), CA3, and CA1 thickness (**C**) in +/+ and hGFAP-Cre-Px1^fl/fl^ mice (*n* = 9 slices from 3 mice for both genotypes). The data underlying this figure can be found in the S1 Metadata J tab.
(PDF)

S4 Fig Conditional and inducible knockout mice for astroglial Px1 display paroxysmal activity. (**A**) Left, representative confocal images from GFAPcreERT2-*mT/mG* mice after TF injection (5 consecutive days) showing recombined astrocytes in green (GFP expression). Scale bar: 20 μm. Right, quantification of the percentage of recombined astrocytes (*n* = 6 slices from 2 mice). (**B**) Left, representative confocal images of Px1 mRNA detected in the CA1 region of the hippocampus by fluorescent in situ hybridisation (FISH by RNAscope) on brain sections from P30 hGFAP-CreERT2-Px1^fl/fl^ mice. Neuron nuclei are immunolabelled with NeuN (top images) and astrocytes with S100β (bottom images). Scale bar: 10 μm. Right, quantification of Px1 mRNA (FISH dot density: dots/mm^2^) in neurons and astrocytes in +/+, hGFAP-Cre-Px1^fl/fl^ and hGFAP-Cre-ERT2-Px1^fl/fl^ mice (*n* = 3, 3, and 3 mice, respectively; one-way ANOVA). (**C-E**) Representative traces of network activity recorded with MEA in +/+ (**C**), hGFAP-CreERT2 (**D**), and hGFAP-Cre-ERT2-Px1^fl/fl^ (**E**) mice after treatment with TF. The corresponding time-frequency plots are shown under the traces. Scale bar: 30 s, 0.2 mV. (**F**) Proportion of bursts and paroxysmal events recorded in TF-treated +/+, hGFAP-CreERT2, and hGFAP-Cre-ERT2-Px1^fl/fl^ mice (+/+ + TF, *n* = 14 slices from 3 mice; hGFAP-CreERT2 + TF, *n* = 26 slices from 4 mice; hGFAP-CreERT2-Px1^fl/fl^ + TF, *n* = 16 slices from 4 mice, Fisher exact test). (**G**) Quantification of bursts and paroxysmal events frequency and duration (+/+ + TF, *n* = 14 slices from 3 mice; hGFAP-CreERT2 + TF, *n* = 26 slices from 4 mice; hGFAP-CreERT2-Px1^fl/fl^ +TF, *n* = 16 slices from 4 mice). Asterisks indicate statistical significance (*p < 0.05). The data underlying this figure can be found in the S1 Metadata K tab.
(PDF)

S5 Fig Global Px1 disruption does not induce paroxysmal activity. (**A**) Representative traces of bursting activity in +/+ mice before (control, Ct) and after ^10^Panx peptide (400 μM, 40 min). Scale bar: 10 s, 200 μV. (**B**) Quantification of burst frequency and duration in +/+ mice before (Ct) and after ^10^Panx peptide (*n* = 5 slices from 3 mice; Student paired *t* test). (**C**) Left, representative confocal images of Px1 mRNA detected in the hippocampus by fluorescent in situ hybridization (FISH by RNAscope) on brain sections from P20-P30 constitutive Px1^−/−^ mice. Neuron nuclei are immunolabeled with NeuN (top images) and astrocytes with S100β (bottom images). Scale bar: 10 μm. Right, quantification of Px1 mRNA (FISH dot density: dots/mm^2^) in neurons and astrocytes in +/+, hGFAP-Cre-Px1^fl/fl^, and constitutive Px1^−/−^ mice (*n* = 3, 3, and 3 mice, respectively, one-way ANOVA). (**D**) Representative traces of bursting activity in +/+ mice (upper trace) and constitutive Px1^−/−^ mice (lower trace). Scale bar: 5 s, 0.1 mV. (**E**) Quantification of burst frequency and duration in +/+ and constitutive Px1^−/−^ hippocampal slices (+/+, *n* = 13 slices from 6 mice; constitutive Px1^−/−^, *n* = 16 slices from 6 mice; Student *t* test). Asterisks indicate statistical significance (*p < 0.05, **p < 0.01). The data underlying this figure can be found in the S1 Metadata L tab.
(PDF)

S6 Fig P2X, P2Y, A2 receptors, KATP, and GIRK channels inhibition does not induce paroxysmal activity in wild-type mice. (**A**) Representative traces of neuronal network activity in +/+ mice before and during application of the P2Y and P2X receptor antagonists PPADS + RB2 (30 μM; *n* = 6 slices from 3 mice), the K_ATP_ antagonist Tolbutamide (Tolb; 500 μM; *n* = 5 slices from 3 mice), the A2R antagonist SCH58261 (100 μM; *n* = 5 slices from 3 mice), and the GIRK channel antagonist SCH23390 (10 μM; *n* = 7 slices from 2 mice). Scale bar, 10 s, 20 μV. (**B**) Quantification of the change in burst frequency in +/+ mice after application of PPADS + RB2, Tolb, and SCH-58261 and SCH23390, normalised to control recordings performed in the same slice before drug application (Student paired *t* test). Asterisks indicate statistical significance (** *p* < 0.01). The data underlying this figure can be found in the S1 Metadata M tab.
(PDF)

S1 Table Quantification of activity patterns. (PDF)

S1 Metadata Underlying data of Figs 1–7 and S1–S6. (XLSX)

## Abbreviations

8-CPE: 8-Cyclopentyl-1,3-dimethylxanthine
ACSF: artificial cerebrospinal fluid
AAV: adeno-associated virus
APs: action potentials
ATP: adenosine triphosphate
CNS: central nervous system
CPA: N6-Cyclopentyladenosine
EEG: electroencephalogram
EtBr: ethidium bromide
fEPSP: field excitatory postsynaptic potential
FISH: fluorescent in situ hybridisation
GFAP: glial fibrillary acidic protein
GTP: guanosine triphosphate
HCN: hyperpolarisation-activated cyclic nucleotide
hGFAP: human glial fibrillary acidic protein
IP: intraperitoneally
ITR: inverted terminal repeat
MEA: multielectrode array
PPADS: pyridoxalphosphate-6-azophenyl-20,40-disulfonic acid
PSD: power spectral density
Px1: Pannexin 1
qPCR: quantitative PCR
RB-2: Reactive Blue-2
TF: tamoxifen
TRAP: Translating Ribosome Affinity Purification
