# Supplementary material for: Pannexin 1 activity in astroglia sets hippocampal neuronal network patterns
Source: PLoS Biol. 2022 Dec 7;20(12):e3001891. doi: 10.1371/journal.pbio.3001891 (PMC9728857; doi:10.1371/journal.pbio.3001891)
Supplement: S1 Fig — (A) Representative astroglial whole-cell current profiles evoked by 150 ms voltage steps (−110 to +100 mV; scale bar, 2 pA, 25 ms) and quantification of current–voltage (I/V) plots and intrinsic membrane properties (B) of astrocytes in +/+ mice before (black trace) and after (red trace) inhibition of Px1 channels with the 10Panx1 peptide (400 μM, 40 min) (n = 4 cells from 3 mice) and in hGFAP-Cre-Px1fl/fl mice (grey trace; n = 5 cells from 3 mice). Asterisks indicate statistical significance (two-way repeated measures ANOVA (A) or Student paired t test (B), ***p < 0.001, **p < 0.01). The data underlying this figure can be found in the S1 Metadata H tab. (PDF) [file pbio.3001891.s001.pdf]

Supporting Figures

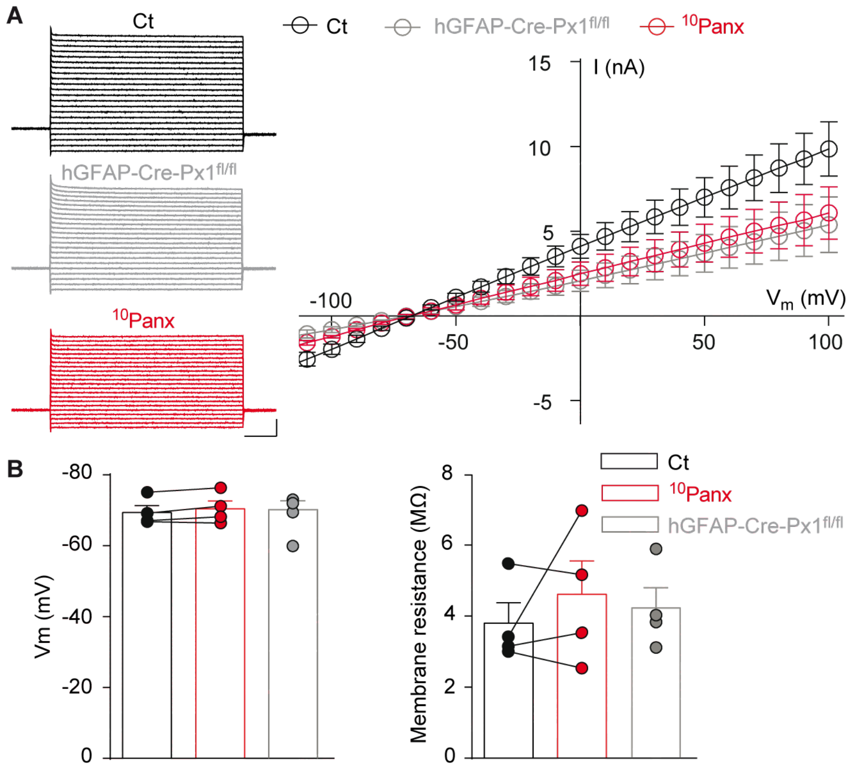

S1 Figure. Pharmacological Px1 inhibition or molecular Px1 disruption decrease astroglial currents at positive potentials. (**A**) Representative astroglial whole-cell current profiles evoked by 150 ms voltage steps (-110 to +100 mV; scale bar, 2 pA, 25 ms) and quantification of current–voltage (I/V) plots and intrinsic membrane properties (**B**) of astrocytes in +/+ mice before (black trace) and after (red trace) inhibition of Px1 channels with the ^{10}Panx1 peptide (400 µM, 40 min) (n = 4 cells from 3 mice) and in hGFAP-Cre-Px1$^{fl/fl}$ mice (grey trace; n = 5 cells from 3 mice). Asterisks indicate statistical significance (two-way repeated measures ANOVA (A) or student's paired *t*-test (B), ****p* < 0.001, ***p* < 0.01). The data underlying this figure can be found in the S1 MetaData H tab.
