# Supplementary material for: Pannexin 1 activity in astroglia sets hippocampal neuronal network patterns
Source: PLoS Biol. 2022 Dec 7;20(12):e3001891. doi: 10.1371/journal.pbio.3001891 (PMC9728857; doi:10.1371/journal.pbio.3001891)
Supplement: S2 Fig — (A) Generation of the hGFAP-Cre-Px1fl/fl mouse (see Materials and methods). (B) Left, representative confocal images of Px1 mRNA in hippocampus by FISH in the hGFAP-Cre-Px1fl/fl mouse. Neuron nuclei are immunolabelled with NeuN (top images) and astrocytes with S100β (bottom images). Scale bar, 10 μm. Right, quantification of Px1 mRNA (FISH dot density: dots/mm2) in neurons and astrocytes in +/+ and hGFAP-Cre-Px1fl/fl mice (n = 3 and 3 mice, respectively; Student t test). (C) EtBr uptake in basal and population activity conditions in +/+ and hGFAP-Cre-Px1fl/fl mice. Scale bar, 20 μm. Insets: zoom on stratum radiatum astrocytes highlighted by a dotted rectangle. Scale bar, 10 μm. s.p., stratum pyramidale; s.r., stratum radiatum. (D) Quantification of neuronal and astroglial EtBr uptake normalised to basal condition in slices from +/+ mice (n = 8 mice) and hGFAP-Cre-Px1fl/fl mice treated or not with 10Panx (n = 14 mice) and scPanx (n = 8 mice; repeated measures one-way ANOVA). Asterisks indicate statistical significance (*p < 0.05, **p < 0.01). The data underlying this figure can be found in the S1 Metadata I tab. (PDF) [file pbio.3001891.s002.pdf]

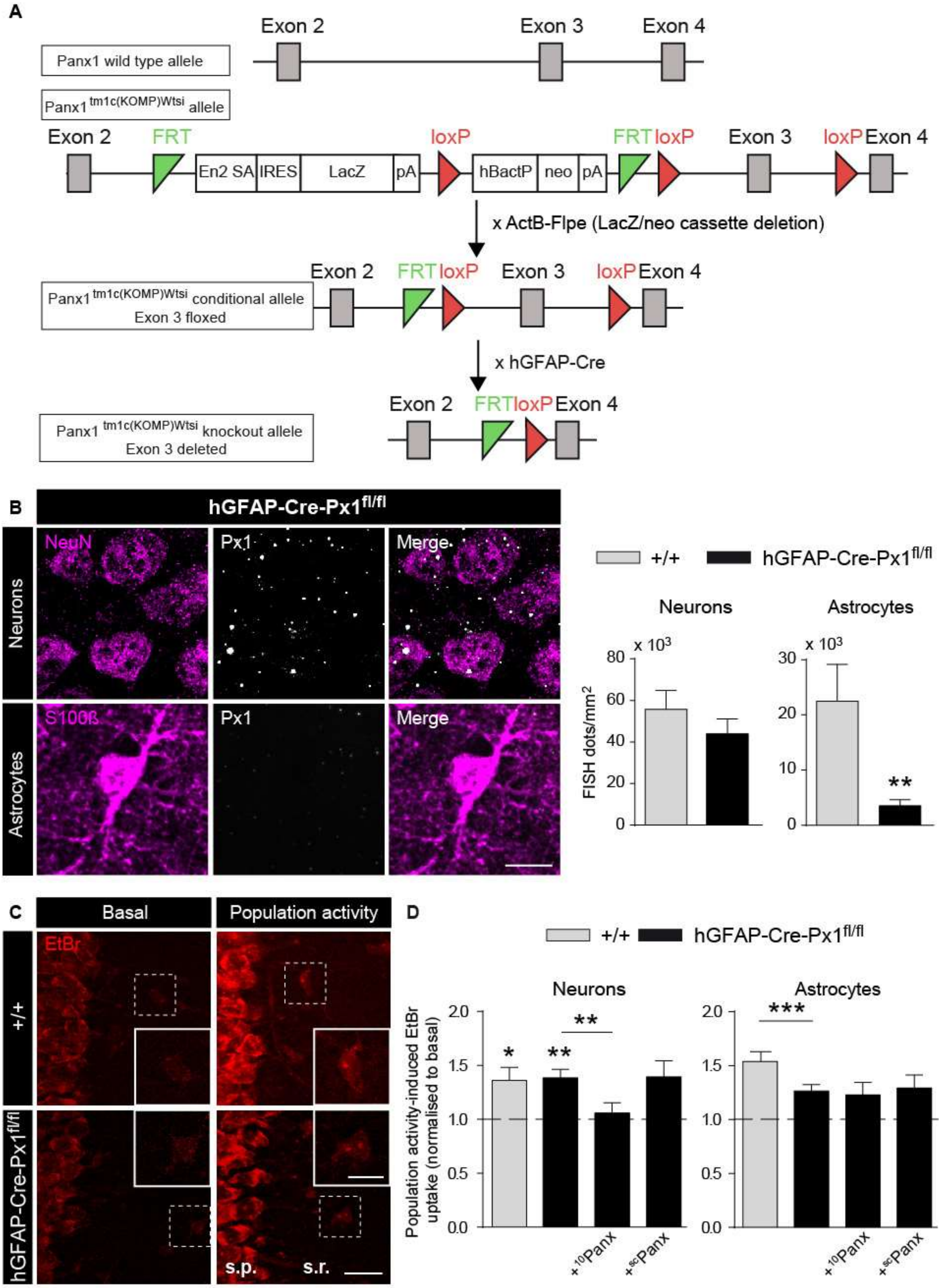
A
Panx1 wild type allele
Exon 2
Exon 3
Exon 4
Panx1 tm1c(KOMP)Wtsi allele
FRT
loxP
En2 SA
IRES
LacZ
pA
hBactP
neo
x ActB-Flpe (LacZ/neo cassette deletion)
Panx1 tm1c(KOMP)Wtsi conditional allele
Exon 3 floxed
x hGFAP-Cre
Panx1 tm1c(KOMP)Wtsi knockout allele
Exon 3 deleted
B
hGFAP-Cre-Px1fl/fl
Neurons
Astrocytes
NeuN
Px1
Merge
S100ß
+/+
FISH dots/mm2
x 10^3
C
Basal
Population activity
EtBr
hGFAP-Cre-Px1fl/fl
s.p.
s.r.
D
Population activity-induced EtBr uptake (normalised to basal)
+10Panx
+scPanx

S2 Figure. Astroglial Px1 expression and function are disrupted in hGFAP-CrePx1$^{fl/fl}$ mouse. (**A**) Generation of the hGFAP-Cre-Px1$^{fl/fl}$ mouse (see material and methods). (**B**) Left, representative confocal images of Px1 mRNA in hippocampus by FISH in the hGFAP-Cre-Px1$^{fl/fl}$ mouse. Neuron nuclei are immunolabelled with NeuN (top images) and astrocytes with S100β (bottom images). Scale bar, 10 µm. Right, quantification of Px1 mRNA (FISH dot density: dots/mm^2) in neurons and astrocytes in +/+ and hGFAP-Cre-Px1$^{fl/fl}$ mice (n = 3 and 3 mice, respectively; student's *t*-test). (**C**) EtBr uptake in Basal and Population activity conditions in wild type (+/+) and hGFAP-Cre-Px1$^{fl/fl}$ mice. Scale bar, 20 µm. Insets: zoom on stratum radiatum astrocytes highlighted by a dotted rectangle. Scale bar, 10 µm. s.p.: *stratum pyramidale*, s.r: *stratum radiatum*. (**D**) Quantification of neuronal and astroglial EtBr uptake normalised to basal condition in slices from wild type mice (n = 8 mice) and hGFAP-Cre-Px1$^{fl/fl}$ mice treated or not with ^{10}Panx (n = 14 mice) and ^{sc}Panx (n = 8 mice; Repeated measures one-way ANOVA). Asterisks indicate statistical significance (*$p < 0.05$, **$p < 0.01$). The data underlying this figure can be found in the S1 MetaData I tab.
