# Supplementary material for: Pannexin 1 activity in astroglia sets hippocampal neuronal network patterns
Source: PLoS Biol. 2022 Dec 7;20(12):e3001891. doi: 10.1371/journal.pbio.3001891 (PMC9728857; doi:10.1371/journal.pbio.3001891)
Supplement: S3 Fig — (A) Immunolabelling of neurons (NeuN, red), astrocytes (GFAP, grey), and nuclei (TO-PRO, blue) in +/+ and hGFAP-Cre-Px1fl/fl mice. Scale bar, 200 μm. (B-C) Quantification of hippocampal neuron and astrocyte cell density (B) and dentate gyrus (DG), CA3, and CA1 thickness (C) in +/+ and hGFAP-Cre-Px1fl/fl mice (n = 9 slices from 3 mice for both genotypes). The data underlying this figure can be found in the S1 Metadata J tab. (PDF) [file pbio.3001891.s003.pdf]

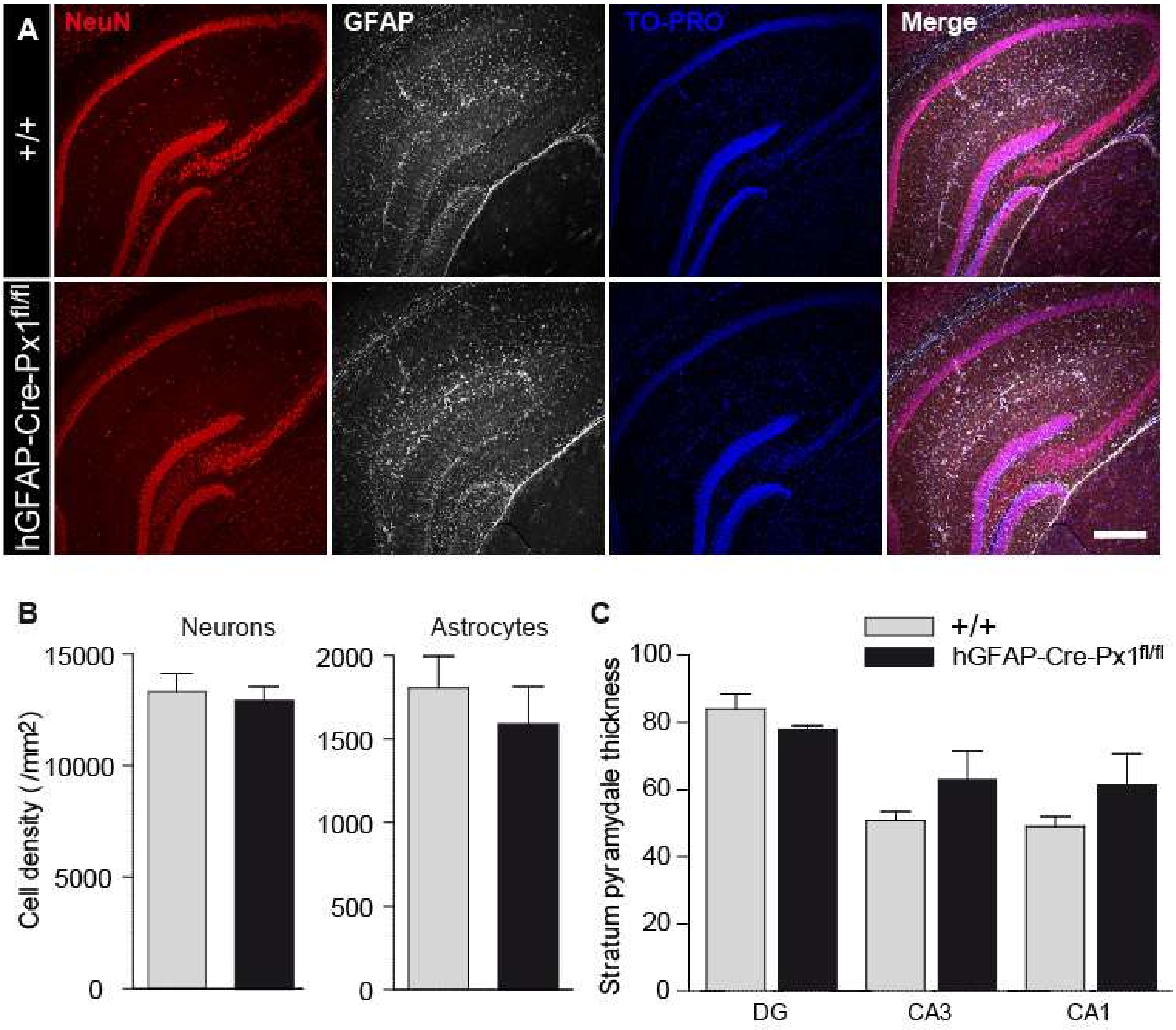

S3 Figure. Astroglial Px1-deleted mice display no gross anatomical alterations and normal hippocampal architecture. (**A**) Immunolabelling of neurons (NeuN, red), astrocytes (GFAP, grey) and nuclei (TO-PRO, blue) in +/+ and hGFAP-Cre-Px1$^{fl/fl}$ mice. Scale bar, 200 µm. (**B-C**) Quantification of hippocampal neuron and astrocyte cell density (**B**) and dentate gyrus (DG), CA3 and CA1 thickness (**C**) in +/+ and hGFAP-Cre-Px1$^{fl/fl}$ mice (n = 9 slices from 3 mice for both genotypes). The data underlying this figure can be found in the S1 MetaData J tab.
