# Supplementary material for: Pannexin 1 activity in astroglia sets hippocampal neuronal network patterns
Source: PLoS Biol. 2022 Dec 7;20(12):e3001891. doi: 10.1371/journal.pbio.3001891 (PMC9728857; doi:10.1371/journal.pbio.3001891)
Supplement: S4 Fig — (A) Left, representative confocal images from GFAPcreERT2-mT/mG mice after TF injection (5 consecutive days) showing recombined astrocytes in green (GFP expression). Scale bar: 20 μm. Right, quantification of the percentage of recombined astrocytes (n = 6 slices from 2 mice). (B) Left, representative confocal images of Px1 mRNA detected in the CA1 region of the hippocampus by fluorescent in situ hybridisation (FISH by RNAscope) on brain sections from P30 hGFAP-CreERT2-Px1fl/fl mice. Neuron nuclei are immunolabelled with NeuN (top images) and astrocytes with S100β (bottom images). Scale bar: 10 μm. Right, quantification of Px1 mRNA (FISH dot density: dots/mm2) in neurons and astrocytes in +/+, hGFAP-Cre-Px1fl/fl and hGFAP-Cre-ERT2-Px1fl/fl mice (n = 3, 3, and 3 mice, respectively; one-way ANOVA). (C-E) Representative traces of network activity recorded with MEA in +/+ (C), hGFAP-CreERT2 (D), and hGFAP-Cre-ERT2-Px1fl/fl (E) mice after treatment with TF. The corresponding time-frequency plots are shown under the traces. Scale bar: 30 s, 0.2 mV. (F) Proportion of bursts and paroxysmal events recorded in TF-treated +/+, hGFAP-CreERT2, and hGFAP-Cre-ERT2-Px1fl/fl mice (+/+ + TF, n = 14 slices from 3 mice; hGFAP-CreERT2 + TF, n = 26 slices from 4 mice; hGFAP-CreERT2-Px1fl/fl + TF, n = 16 slices from 4 mice, Fisher exact test). (G) Quantification of bursts and paroxysmal events frequency and duration (+/+ + TF, n = 14 slices from 3 mice; hGFAP-CreERT2 + TF, n = 26 slices from 4 mice; hGFAP-CreERT2-Px1fl/fl +TF, n = 16 slices from 4 mice). Asterisks indicate statistical significance (*p < 0.05). The data underlying this figure can be found in the S1 Metadata K tab. (PDF) [file pbio.3001891.s004.pdf]

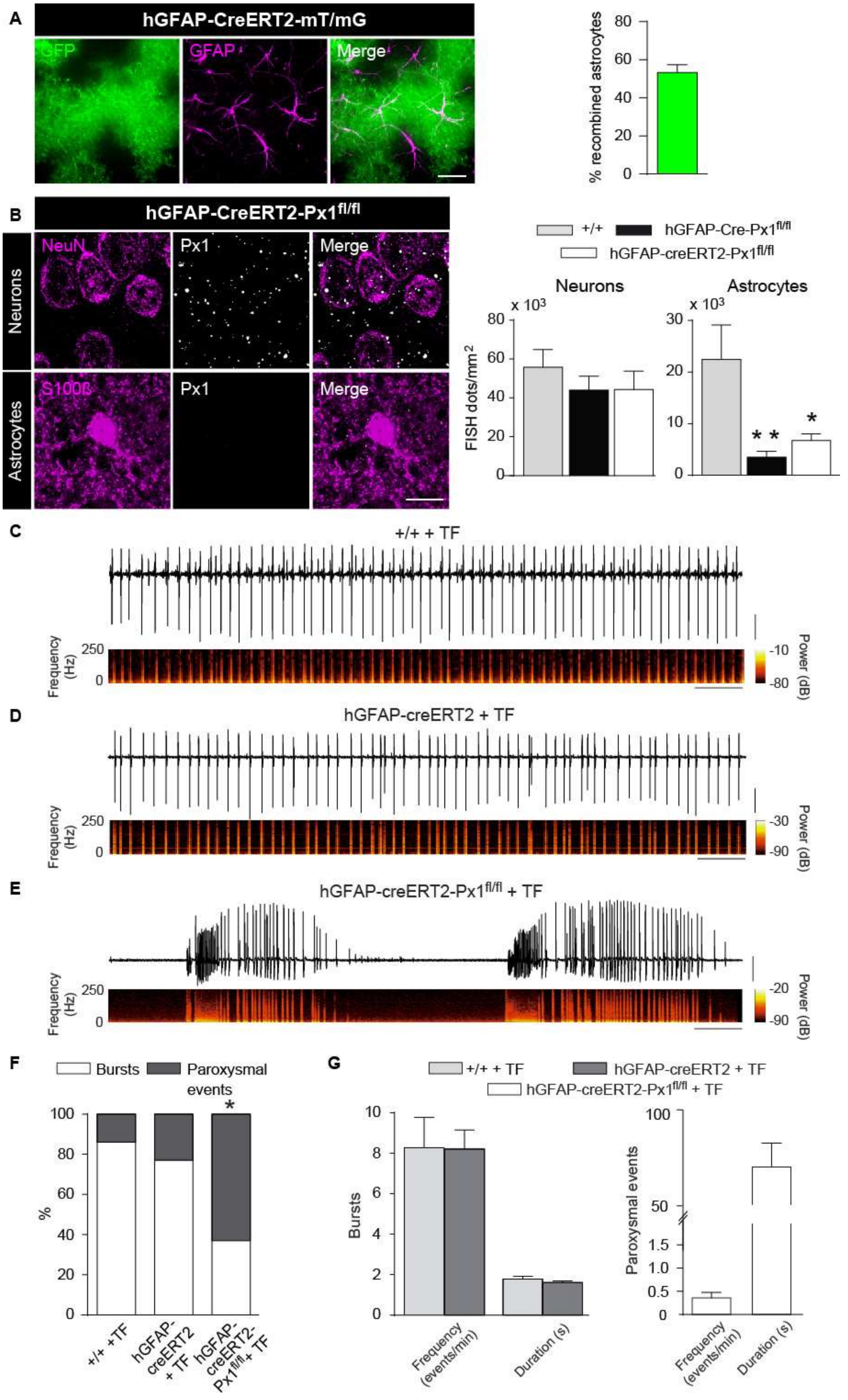
A
hGFAP-CreERT2-mT/mG
GFP
GFAP
Merge
% recombined astrocytes
B
hGFAP-CreERT2-Px1fl/fl
Neurons
Astrocytes
NeuN
Px1
Merge
S100ß
+/+
hGFAP-Cre-Px1fl/fl
hGFAP-creERT2-Px1fl/fl
Neurons
Astrocytes
FISH dots/mm2
x 10³
C
+/+ + TF
Frequency (Hz)
Power (dB)
D
hGFAP-creERT2 + TF
E
hGFAP-creERT2-Px1fl/fl + TF
F
Bursts
Paroxysmal events
%
+/+ +TF
hGFAP-creERT2 + TF
hGFAP-creERT2-Px1fl/fl + TF
G
+/+ + TF
hGFAP-creERT2 + TF
hGFAP-creERT2-Px1fl/fl + TF
Bursts
Paroxysmal events
Frequency (events/min)
Duration (s)

S4 Figure. Conditional and inducible knockout mice for astroglial Px1 display paroxysmal activity. (**A**) Left, representative confocal images from GFAPcreERT2-*mT/mG* mice after TF injection (5 consecutive days) showing recombined astrocytes in green (GFP expression). Scale bar: 20 µm. Right, quantification of the percentage of recombined astrocytes (n = 6 slices from 2 mice). (**B**) Left, representative confocal images of Px1 mRNA detected in the CA1 region of the hippocampus by fluorescent *in situ* hybridisation (FISH by RNAscope) on brain sections from P20-P30 hGFAP-CreERT2-Px1$^{fl/fl}$ mice. Neuron nuclei are immunolabelled with NeuN (top images) and astrocytes with S100β (bottom images). Scale bar: 10 µm. Right, quantification of Px1 mRNA (FISH dot density: dots/mm^2) in neurons and astrocytes in +/+, hGFAP-Cre-Px1$^{fl/fl}$ and hGFAP-Cre-ERT2-Px1$^{fl/fl}$ mice (n = 3, 3 and 3 mice, respectively; one-way ANOVA). (**C-E**) Representative traces of network activity recorded with MEA in +/+ (**C**), hGFAP-CreERT2 (**D**) and hGFAP-Cre-ERT2-Px1$^{fl/fl}$ (**E**) mice after treatment with tamoxifen (TF). The corresponding time-frequency plots are shown under the traces. Scale bar: 30 sec, 0.2 mV. (**F**) Proportion of bursts and paroxysmal events recorded in TF-treated +/+, hGFAP-CreERT2 and hGFAP-Cre-ERT2-Px1$^{fl/fl}$ mice (+/+ + TF, n = 14 slices from 3 mice; hGFAP-CreERT2 + TF, n = 26 slices from 4 mice; hGFAP-CreERT2-Px1$^{fl/fl}$ + TF, n = 16 slices from 4 mice, Fisher's exact test). (**G**) Quantification of bursts and paroxysmal events frequency and duration (+/+ + TF, n = 14 slices from 3 mice; hGFAP-CreERT2 + TF, n = 26 slices from 4 mice; hGFAP-CreERT2-Px1$^{fl/fl}$ +TF, n = 16 slices from 4 mice). Asterisks indicate statistical significance (*$p < 0.05$). The data underlying this figure can be found in the S1 MetaData K tab.
