# Supplementary material for: Pannexin 1 activity in astroglia sets hippocampal neuronal network patterns
Source: PLoS Biol. 2022 Dec 7;20(12):e3001891. doi: 10.1371/journal.pbio.3001891 (PMC9728857; doi:10.1371/journal.pbio.3001891)
Supplement: S5 Fig — (A) Representative traces of bursting activity in +/+ mice before (control, Ct) and after 10Panx peptide (400 μM, 40 min). Scale bar: 10 s, 200 μV. (B) Quantification of burst frequency and duration in +/+ mice before (Ct) and after 10Panx peptide (n = 5 slices from 3 mice; Student paired t test). (C) Left, representative confocal images of Px1 mRNA detected in the hippocampus by fluorescent in situ hybridization (FISH by RNAscope) on brain sections from P20-P30 constitutive Px1−/− mice. Neuron nuclei are immunolabeled with NeuN (top images) and astrocytes with S100β (bottom images). Scale bar: 10 μm. Right, quantification of Px1 mRNA (FISH dot density: dots/mm2) in neurons and astrocytes in +/+, hGFAP-Cre-Px1fl/fl, and constitutive Px1−/− mice (n = 3, 3, and 3 mice, respectively, one-way ANOVA). (D) Representative traces of bursting activity in +/+ mice (upper trace) and constitutive Px1−/− mice (lower trace). Scale bar: 5 s, 0.1 mV. (E) Quantification of burst frequency and duration in +/+ and constitutive Px1−/− hippocampal slices (+/+, n = 13 slices from 6 mice; constitutive Px1−/−, n = 16 slices from 6 mice; Student t test). Asterisks indicate statistical significance (*p < 0.05, **p < 0.01). The data underlying this figure can be found in the S1 Metadata L tab. (PDF) [file pbio.3001891.s005.pdf]

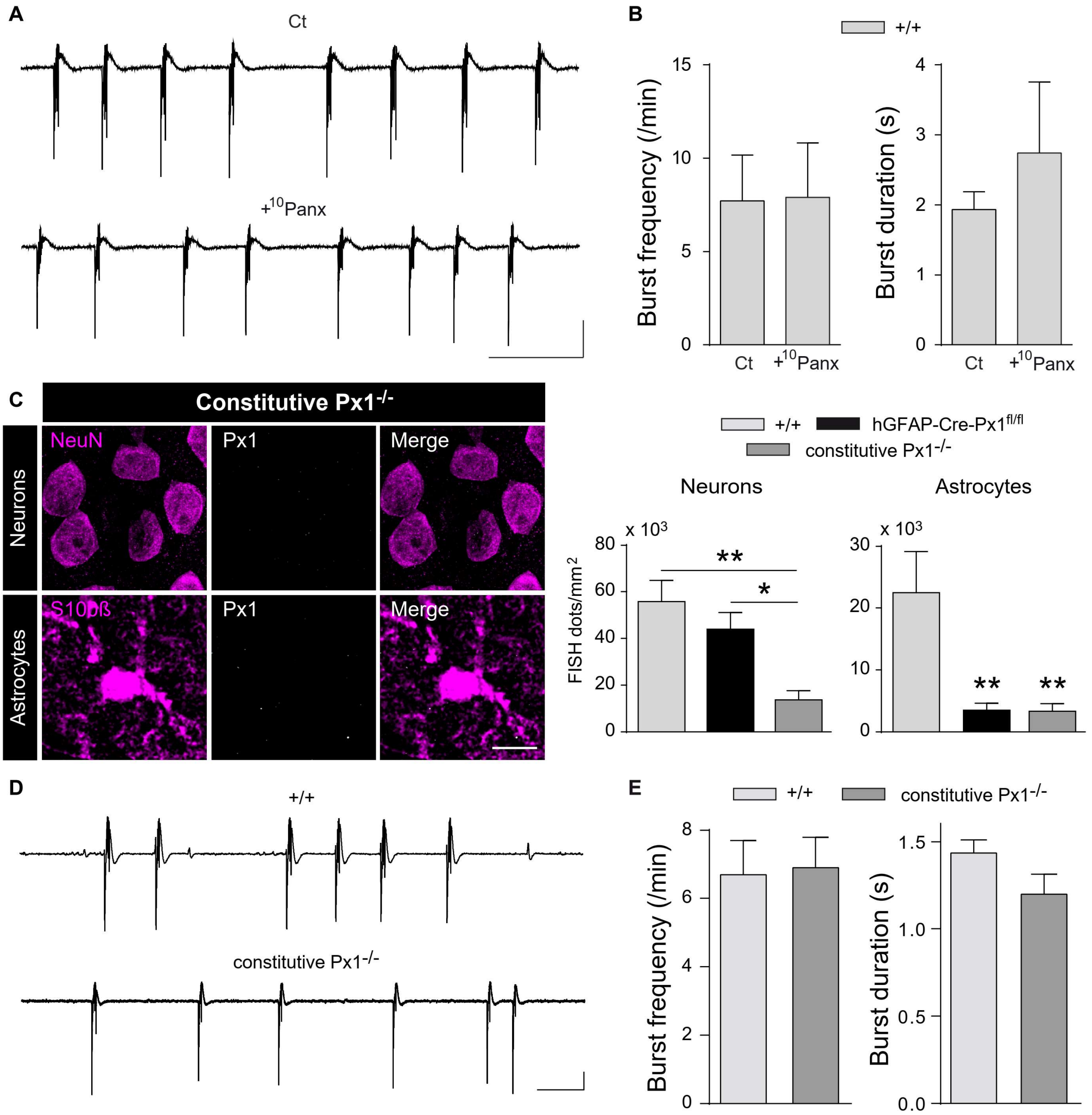

S5 Figure. Global Px1 disruption does not induce paroxysmal activity. (**A**) Representative traces of bursting activity in +/+ mice before (control, Ct) and after ^{10}Panx peptide (400 µM, 40 min). Scale bar: 10 s, 200 µV. (**B**) Quantification of burst frequency and duration in +/+ mice before (Ct) and after ^{10}Panx peptide (n = 5 slices from 3 mice; student's paired *t*-test). (**C**) Left, representative confocal images of Px1 mRNA detected in the hippocampus by fluorescent *in situ* hybridization (FISH by RNAscope) on brain sections from P20-P30 constitutive Px1$^{-/-}$ mice. Neuron nuclei are immunolabeled with NeuN (top images) and

astrocytes with S100β (bottom images). Scale bar: 10 µm. Right, quantification of Px1 mRNA (FISH dot density: dots/mm^2) in neurons and astrocytes in +/+, hGFAP-Cre-Px1$^{fl/fl}$ and constitutive Px1$^{-/-}$ mice (n = 3, 3 and 3 mice respectively, one-way ANOVA). (**D**) Representative traces of bursting activity in +/+ mice (upper trace) and constitutive Px1$^{-/-}$ mice (lower trace). Scale bar: 5 s, 0.1 mV. (**E**) Quantification of burst frequency and duration in +/+ and constitutive Px1-/- hippocampal slices (+/+, n = 13 slices from 6 mice; constitutive Px1$^{-/-}$, n = 16 slices from 6 mice; student's *t*-test). Asterisks indicate statistical significance (*$p < 0.05$, **$p < 0.01$). The data underlying this figure can be found in the S1 MetaData L tab.
