# Supplementary material for: Pannexin 1 activity in astroglia sets hippocampal neuronal network patterns
Source: PLoS Biol. 2022 Dec 7;20(12):e3001891. doi: 10.1371/journal.pbio.3001891 (PMC9728857; doi:10.1371/journal.pbio.3001891)
Supplement: S6 Fig — (A) Representative traces of neuronal network activity in +/+ mice before and during application of the P2Y and P2X receptor antagonists PPADS + RB2 (30 μM; n = 6 slices from 3 mice), the KATP antagonist Tolbutamide (Tolb; 500 μM; n = 5 slices from 3 mice), the A2R antagonist SCH58261 (100 μM; n = 5 slices from 3 mice), and the GIRK channel antagonist SCH23390 (10 μM; n = 7 slices from 2 mice). Scale bar, 10 s, 20 μV. (B) Quantification of the change in burst frequency in +/+ mice after application of PPADS + RB2, Tolb, and SCH-58261 and SCH23390, normalised to control recordings performed in the same slice before drug application (Student paired t test). Asterisks indicate statistical significance (** p < 0.01). The data underlying this figure can be found in the S1 Metadata M tab. (PDF) [file pbio.3001891.s006.pdf]

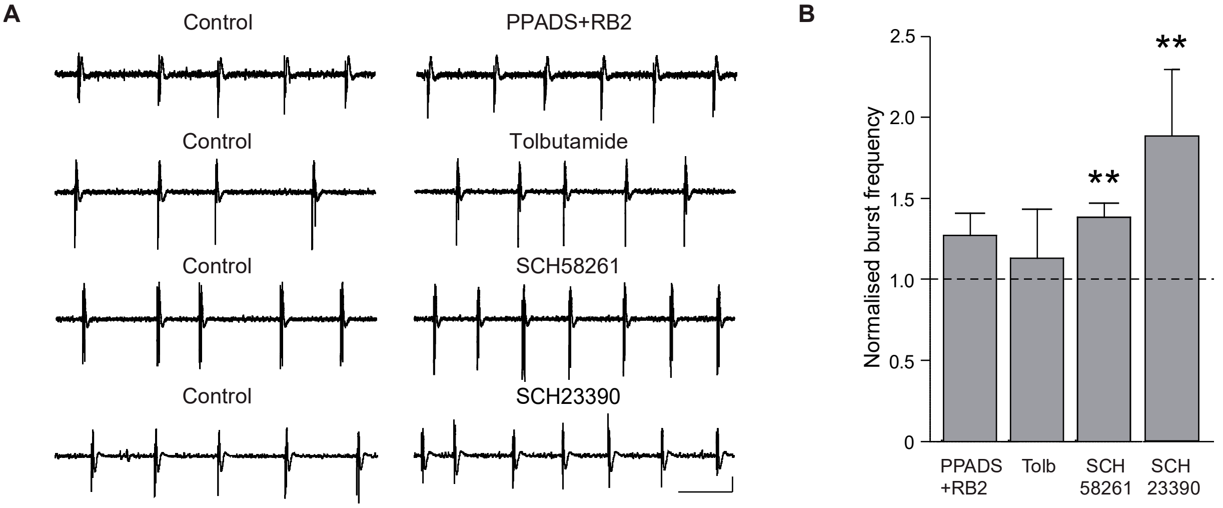

S6 Figure. P2X, P2Y, A2 receptors, KATP and GIRK channels inhibition does not induce paroxysmal activity in wild-type mice. (**A**) Representative traces of neuronal network activity in +/+ mice before and during application of the P2Y and P2X receptor antagonists PPADS + RB2 (30 µM; n = 6 slices from 3 mice), the KATP antagonist Tolbutamide (Tolb; 500 µM; n = 5 slices from 3 mice), the A2R antagonist SCH58261 (100 µM; n = 5 slices from 3 mice) and the GIRK channel antagonist SCH23390 (10 µM; n = 7 slices from 2 mice). Scale bar, 10 sec, 20 µV. (**B**) Quantification of the change in burst frequency in +/+ mice after application of PPADS + RB2, Tolb and SCH-58261 and SCH23390, normalised to control recordings performed in the same slice before drug application (student's paired *t*-test). Asterisks indicate statistical significance (** $p < 0.01$). The data underlying this figure can be found in the S1 MetaData M tab.
