# Supplementary material for: Pannexin 1 activity in astroglia sets hippocampal neuronal network patterns
Source: PLoS Biol. 2022 Dec 7;20(12):e3001891. doi: 10.1371/journal.pbio.3001891 (PMC9728857; doi:10.1371/journal.pbio.3001891)
Supplement: S1 Table — (PDF) [file pbio.3001891.s007.pdf]

Supporting Table 1: Quantification of activity patterns

	Paroxysmal events			Bursts		
	**% slices with paroxysmal events**	**Frequency (/min)**	**Duration (s)**	**% slices with bursts**	**Frequency (/min)**	**Duration (s)**
**Fig 5B: +/+** (n = 9 slices from 3 mice)						
Control	0	Nd	Nd	100	4.55 ± 0.47	1.66 ± 0.55
+ 8-CPT 1 µM	100	n/a	n/a	0	Nd	Nd
**Fig 5B: A1R-/-** (n = 10 slices from 3 mice)						
Control	70	n/a	n/a	30	9.73 ± 1.39	1.30 ± 0.10
**Fig 6B: hGFAP-Cre-Px1$^{fl/fl}$ + AAV-GFAP-GFP-Px1** (n = 21 slices from 4 mice)						
Control	19,1	0.73 ± 0.29	54.44 ± 16.12	80.9	6.05 ± 0.64	1.86 ± 0.10
**Fig 6E: hGFAP-Cre-Px1$^{fl/fl}$** (n = 6 slices from 3 mice)						
Control	100	0.83 ± 0.27	53.13 ± 16.74	0	Nd	Nd
+ CPA 300 nM	0	Nd	Nd	100	3.39 ± 2.81	1.34 ± 0.28
**Fig 7C: hGFAP-Cre-Px1$^{fl/fl}$** (n = 5 slices from 4 mice)						
Control	100	0.87 ± 0.6	69.57 ± 24.4	0	Nd	Nd
+ ZD7288 10 µM	0	Nd	Nd	100	2.26 ± 0.6	1.72 ± 0.22
**Fig 7D: +/+** (n = 6 slices from 2 mice)						
Control	0	Nd	Nd	100	4.66 ± 0.89	1.41 ± 0.14
+ ZD7288 10 µM	0	Nd	Nd	100	3.67 ± 1.16	1.53 ± 0.08
**Fig 7F: +/+** (n = 5 slices from 4 mice)						
Control	0	Nd	Nd	100	8.42 ± 2.08	1.76 ± 0.17
+ 8-CPT 1 µM	100	n/a	n/a	0	Nd	Nd
+ ZD7288 10 µM	0	Nd	Nd	100	10.92 ± 1.27	1.51 ± 0.14
**S4 Fig: +/+ + TF** (n = 14 slices from 3 mice)						
Control	14.3	0.20 ± 0.02	104.55±30.69	85.7	8.27 ± 1.51	1.78 ± 0.13
**S4 Fig: hGFAP-CreERT2 + TF** (n = 26 slices from 4 mice)						
Control	23	0.20 ± 0.03	85.24 ±15.46	77	8.22 ± 0.94	1.61 ± 0.09
**S4 Fig: hGFAP-CreERT2-Px1$^{fl/fl}$ + TF** (n = 16 slices from 4 mice)						
Control	62.5	0.40 ± 0.12	68.08 ± 12	37.5	6.53 ± 1.27	1.46 ± 0.15
**S5 Fig: +/+** (n = 5 slices from 3 mice)						
Control	0	Nd	Nd	100	7.72 ± 2.45	1.94 ± 0.25
+ ^{10}Panx	0	Nd	Nd	100	7.91 ± 2.91	2.74 ± 1.01

*Nd = not detected.
*n/a = not applicable
